# Supplementary material for: Barriers and facilitators to dementia care in long-term care facilities: protocol for a qualitative systematic review and meta-synthesis
Source: BMJ Open. 2023 Nov 1;13(11):e076058. doi: 10.1136/bmjopen-2023-076058 (PMC10626821; doi:10.1136/bmjopen-2023-076058)
Supplement: Supplementary data [file bmjopen-2023-076058supp001.pdf]

Barriers and facilitators to dementia care in long-term care facilities：A systematic review and meta-synthesis protocol

Supplement file 1 - Searched databases and search strategies

1. Search strategy for Web of Science	
#1	**TI=**(dement* OR "presenile dementia" OR "cognitive impairment" OR "cognitive dysfunction" OR "Alzheimer's disease" OR "Alzheimer's dementia" OR AD OR "mild cognitive impairment" OR "huntington disease" OR "lewy body" OR "chronic cerebrovascular" OR "parkinson disease dementia" OR "parkinson dementia" OR “lewy bodies” OR “lewy neurites” OR “dementia, vascular” OR “vascular dementia*”) OR **AB=**(dementia OR "presenile dementia" OR "cognitive impairment" OR "cognitive dysfunction" OR "Alzheimer's disease" OR "Alzheimer's dementia" OR AD OR "mild cognitive impairment" OR "huntington disease" OR "lewy body" OR "chronic cerebrovascular" OR "parkinson disease dementia" OR "parkinson dementia" OR “lewy bodies” OR “lewy neurites” OR “dementia, vascular” OR “vascular dementia*”)
#2	**TI=**(nursing OR "practical nursing" OR "care, nursing" OR "management, nursing Care" OR "care, long-term" OR "long term care" OR care OR caring OR caregiving OR manag* OR "long-term care") OR **AB=**(nursing OR "practical nursing" OR "care, nursing" OR "management, nursing Care" OR "care, long-term" OR "long term care" OR care OR caring OR caregiving OR manag* OR "long-term care")
#3	**TI=**("elderly care institution" OR "nursing home*" OR "health care setting" OR "long term care facilities" OR "residential aged care" OR "care home*" OR "residential facilities" OR "homes for aged" OR "residential home*" OR "residential facilit*" OR "nursing home facility*" OR "nursing home care" OR "nursing home placement" OR "old people's home*" OR "homes for the aged" OR "old age home" OR "old age homes" OR "housing for the elderly") OR **AB=**("elderly care institution" OR "nursing home*" OR "health care setting" OR "long term care facilities" OR "residential aged care" OR "care home*" OR "residential facilities" OR "homes for aged" OR "residential home*" OR "residential facilit*" OR "nursing home facility*" OR "nursing home care" OR "nursing home placement" OR "old people's home*" OR "homes for the aged" OR "old age home" OR "old age homes" OR "housing for the elderly")
#4	**TI=**("nursing methodology" OR "case study" OR "constant comparison" OR "content analysis" OR "descriptive study" OR "discourse analysis" OR ethnography OR exploratory OR feminist OR "focus group*" OR "grounded theory" OR hermeneutic OR interview OR narrative OR naturalistic OR "participant observation" OR phenomenology OR "qualitative method" OR "qualitative research" OR "qualitative study" OR "thematic analysis" OR "mixed method research" OR "mixed methods research") OR **AB=**("nursing methodology" OR "case study" OR "constant comparison" OR "content analysis" OR "descriptive study" OR "discourse analysis" OR ethnography OR exploratory OR feminist OR "focus group*" OR "grounded theory" OR hermeneutic OR interview OR narrative OR naturalistic OR "participant observation" OR phenomenology OR "qualitative method" OR "qualitative research" OR

	"qualitative study" OR "thematic analysis" OR "mixed method research" OR "mixed methods research")
#5	#1 AND #2 AND #3 AND #4
2. Search strategy for Embase	
#1	**'Alzheimer disease'/exp OR 'dementia'/exp OR 'mild cognitive impairment'/exp OR 'Lewy body'/exp OR 'multiinfarct dementia'/exp**
#2	dement*:**ti,ab** OR 'presenile dementia':ti,ab OR 'cognitive impairment':ti,ab OR alzheimer*:ti,ab OR ad:ti,ab OR 'mild cognitive impairment':ti,ab OR 'huntington disease':ti,ab OR 'lewy body':ab OR 'chronic cerebrovascular':ti,ab OR 'parkinson disease dementia':ti,ab OR 'parkinson dementia':ti,ab OR 'cognitive dysfunction':ti,ab OR 'lewy bodies':ti,ab OR 'lewy neurites':ti,ab OR 'dementia, vascular':ti,ab OR 'multiinfarct dementia': ti,ab
#3	#1 OR #2
#4	**'management'/exp OR 'nursing'/exp OR 'practical nursing'/exp OR 'care'/exp OR 'custodial care'/exp OR 'nursing care'/exp OR 'long term care'/exp**
#5	nursings:**ti,ab** OR 'practical nursing':ti,ab OR 'nursing care':ti,ab OR 'management, nursing care':ti,ab OR 'care, long-term':ti,ab OR 'long term care':ti,ab OR care:ti,ab OR caring:ti,ab OR caregiving:ti,ab OR manag*:ti,ab OR 'long-term care':ti,ab
#6	#4 OR #5
#7	**'nursing home'/exp OR 'residential home'/exp OR 'home for the aged'/exp**
#8	'elderly care institution':**ti,ab** OR 'health care setting':ti,ab OR 'long term care facilities':ti,ab OR 'residential aged care':ti,ab OR 'care home*':ti,ab OR 'nursing home*':ti,ab OR 'residential facilities':ti,ab OR 'homes for aged':ti,ab OR 'residential home*':ti,ab OR 'residential facilit*':ti,ab OR 'nursing home facility*':ti,ab OR 'nursing home care':ti,ab OR 'nursing home placement':ti,ab OR 'homes for the aged':ti,ab OR 'old age home':ti,ab OR 'old age homes':ti,ab OR 'housing for the elderly':ti,ab
#9	#7 OR #8
#10	**'qualitative research'/exp**
#11	'nursing methodology':**ti,ab** OR 'case study':ti,ab OR 'constant comparison':ti,ab OR 'content analysis':ti,ab OR 'descriptive study':ti,ab OR 'discourse analysis':ti,ab OR ethnography:ti,ab OR exploratory:ti,ab OR feminist:ti,ab OR 'focus group*':ti,ab OR 'grounded theory':ti,ab OR hermeneutic:ti,ab OR interview:ti,ab OR narrative:ti,ab OR naturalistic:ti,ab OR 'participant observation':ti,ab OR phenomenology:ti,ab OR 'qualitative method':ti,ab OR 'qualitative research':ti,ab OR 'qualitative study':ti,ab OR 'thematic analysis':ti,ab OR 'mixed method research':ti,ab OR 'mixed methods research':ti,ab
#12	#10 OR #11
#13	#3 AND #6 AND #9 AND #12 AND
3. Search strategy for Medline	
#1	**exp Dementia/ or exp Frontotemporal Dementia/ or exp Dementia, Vascular/ or Cognitive Dysfunction/ or exp Lewy Bodies/ or exp Parkinson Disease/**
#2	(dement* or "presenile dementia" or "cognitive impairment" or "cognitive dysfunction" or "Alzheimer's disease" or "Alzheimer's dementia" or AD or "mild cognitive impairment" or "huntington disease" or "lewy body" or "chronic cerebrovascular" or "parkinson disease dementia" or "parkinson dementia" or "parkinson dementia").tw.

#3	#1 or #2
#4	**exp Nursing/ or exp Nursing, Practical/ or exp Custodial Care/ or Nursing Care/ or exp Long-Term Care/**
#5	(nursing or "practical nursing" or "care, nursing" or "management, nursing Care" or "care, long-term" or "long term care" or care or caring or caregiving or manag* or "long-term care").tw.
#6	#4 or #5
#7	**exp Nursing Homes/ or exp Residential Facilities/ or Homes for the Aged/**
#8	("elderly care institution" or "nursing home*" or "health care setting" or "long term care facilities" or "residential aged care" or "care home*" or "residential facilities" or "homes for aged" or "residential home*" or "residential facilit*" or "nursing home facility*" or "nursing home care" or "nursing home placement" or "old people's home*" or "homes for the aged" or "old age home" or "old age homes" or "housing for the elderly").tw.
#9	#7 or #8
#10	**exp Qualitative Research/**
#11	("nursing methodology" or "case study" or "constant comparison" or "content analysis" or "descriptive study" or "discourse analysis" or ethnography or exploratory or feminist or "focus group*" or "grounded theory" or hermeneutic or interview or narrative or naturalistic or "participant observation" or phenomenology or "qualitative method" or "qualitative research" or "qualitative study" or "thematic analysis" or "mixed method research" or "mixed methods research").tw.
#12	#10 or #11
#13	#3 and #6 and #9 and #12
4. Search strategy for CINAHL	
#1	**(MH "Dementia+") OR (MH "Frontotemporal Dementia+") OR (MH "Dementia, Vascular+") OR (MH "Lewy Body Disease") OR (MH "Mild Cognitive Impairment")**
#2	**TI** (dement* OR "presenile dementia" OR "cognitive impairment" OR "cognitive dysfunction" OR "Alzheimer's disease" OR "Alzheimer's dementia" OR AD OR "mild cognitive impairment" OR "huntington disease" OR "lewy body" OR "chronic cerebrovascular" OR "parkinson disease dementia" OR "parkinson dementia" OR “lewy bodies” OR “lewy neurites” OR “dementia, vascular” OR “vascular dementia*”)
#3	**AB** (dement* OR "presenile dementia" OR "cognitive impairment" OR "cognitive dysfunction" OR "Alzheimer's disease" OR "Alzheimer's dementia" OR AD OR "mild cognitive impairment" OR "huntington disease" OR "lewy body" OR "chronic cerebrovascular" OR "parkinson disease dementia" OR "parkinson dementia" OR “lewy bodies” OR “lewy neurites” OR “dementia, vascular” OR “vascular dementia*”)
#4	#1 OR #2 OR #3
#5	**(MH "Caring+") OR (MH "Long Term Care") OR (MH "Management+")**
#6	**TI** (nursing OR "practical nursing" OR "care, nursing" OR "management, nursing Care" OR "care, long-term" OR "long term care" OR care OR caring OR caregiving OR manag* OR "long-term care")
#7	**AB** (nursing OR "practical nursing" OR "care, nursing" OR "management, nursing Care" OR "care, long-term" OR "long term care" OR care OR caring OR caregiving OR manag* OR "long-term care")

#8	#5 OR #6 OR #7
#9	**(MH "Nursing Homes+") OR (MH "Residential Facilities+")**
#10	**TI** ("elderly care institution" OR "nursing home*" OR "health care setting" OR "long term care facilities" OR "residential aged care" OR "care home*" OR "residential facilities" OR "homes for aged" OR "residential home*" OR "residential facilit*" OR "nursing home facility*" OR "nursing home care" OR "nursing home placement" OR "old people's home*" OR "homes for the aged" OR "old age home" OR "old age homes" OR "housing for the elderly")
#11	**AB** ("elderly care institution" OR "nursing home*" OR "health care setting" OR "long term care facilities" OR "residential aged care" OR "care home*" OR "residential facilities" OR "homes for aged" OR "residential home*" OR "residential facilit*" OR "nursing home facility*" OR "nursing home care" OR "nursing home placement" OR "old people's home*" OR "homes for the aged" OR "old age home" OR "old age homes" OR "housing for the elderly")
#12	#9 OR #10 OR #11
#13	**(MH "Qualitative Studies+")**
#14	**TI** ("nursing methodology" OR "case study" OR "constant comparison" OR "content analysis" OR "descriptive study" OR "discourse analysis" OR ethnography OR exploratory OR feminist OR "focus group*" OR "grounded theory" OR hermeneutic OR interview OR narrative OR naturalistic OR "participant observation" OR phenomenology OR "qualitative method" OR "qualitative research" OR "qualitative study" OR "thematic analysis" OR "mixed method research" OR "mixed methods research")
#15	**AB** ("nursing methodology" OR "case study" OR "constant comparison" OR "content analysis" OR "descriptive study" OR "discourse analysis" OR ethnography OR exploratory OR feminist OR "focus group*" OR "grounded theory" OR hermeneutic OR interview OR narrative OR naturalistic OR "participant observation" OR phenomenology OR "qualitative method" OR "qualitative research" OR "qualitative study" OR "thematic analysis" OR "mixed method research" OR "mixed methods research")
#16	#13 OR #14 OR #15
#17	#4 AND #8 AND #12 AND #16
5. Search strategy for PsycINFO	
#1	**(DE "Dementia") OR (DE "Dementia with Lewy Bodies") OR (DE "Vascular Dementia") OR (DE "Alzheimer's Disease") OR (DE "Cognitive Impairment") OR (DE "Mild Cognitive Impairment")**
#2	**TI** (dement* OR "presenile dementia" OR "cognitive impairment" OR "cognitive dysfunction" OR "Alzheimer's disease" OR "Alzheimer's dementia" OR "AD" OR "mild cognitive impairment" OR "huntington disease" OR "lewy body" OR "chronic cerebrovascular" OR "parkinson disease dementia" OR "parkinson dementia" OR “lewy bodies” OR “lewy neurites” OR “dementia, vascular” OR “vascular dementia*”)
#3	**AB** (dement* OR "presenile dementia" OR "cognitive impairment" OR "cognitive dysfunction" OR "Alzheimer's disease" OR "Alzheimer's dementia" OR "AD" OR "mild cognitive impairment" OR "huntington disease" OR "lewy body" OR "chronic cerebrovascular" OR "parkinson disease dementia" OR "parkinson dementia" OR “lewy bodies” OR “lewy neurites” OR “dementia, vascular” OR “vascular dementia*”)

#4	#1 OR #2 OR #3
#5	**(DE "Nursing") OR (DE "Caregiving") OR (DE "Long Term Care") OR (DE "Management")**
#6	**TI** (nursing OR "practical nursing" OR "care, nursing" OR "management, nursing Care" OR "care, long-term" OR "long term care" OR care OR caring OR caregiving OR cargiving OR manag* OR "long-term care")
#7	**AB** (nursing OR "practical nursing" OR "care, nursing" OR "management, nursing Care" OR "care, long-term" OR "long term care" OR care OR caring OR caregiving OR manag* OR "long-term care")
#8	#5 OR #6 OR #7
#9	**(DE "Nursing Homes") OR (DE "Residential Care Institutions")**
#10	**TI** ("elderly care institution" OR "nursing home*" OR "health care setting" OR "long term care facilities" OR "residential aged care" OR "care home*" OR "residential facilities" OR "homes for aged" OR "residential home*" OR "residential facilit*" OR "nursing home facility*" OR "nursing home care" OR "nursing home placement" OR "old people's home*" OR "homes for the aged" OR "old age home" OR "old age homes" OR "housing for the elderly")
#11	**AB** ("elderly care institution" OR "nursing home*" OR "health care setting" OR "long term care facilities" OR "residential aged care" OR "care home*" OR "residential facilities" OR "homes for aged" OR "residential home*" OR "residential facilit*" OR "nursing home facility*" OR "nursing home care" OR "nursing home placement" OR "old people's home*" OR "homes for the aged" OR "old age home" OR "old age homes" OR "housing for the elderly")
#12	#9 OR #10 OR #11
#13	**DE "Focus Group" OR DE "Focus Group Interview" OR DE "Qualitative Methods" OR DE "Grounded Theory" OR DE "Interpretative Phenomenological Analysis" OR DE "Narrative Analysis" OR DE "Semi-Structured Interview" OR DE "Thematic Analysis"**
#14	**TI** ("nursing methodology" OR "case study" OR "constant comparison" OR "content analysis" OR "descriptive study" OR "discourse analysis" OR ethnography OR exploratory OR feminist OR "focus group*" OR "grounded theory" OR hermeneutic OR interview OR narrative OR naturalistic OR "participant observation" OR phenomenology OR "qualitative method" OR "qualitative research" OR "qualitative study" OR "thematic analysis" OR "mixed method research" OR "mixed methods research")
#15	**AB** ("nursing methodology" OR "case study" OR "constant comparison" OR "content analysis" OR "descriptive study" OR "discourse analysis" OR ethnography OR exploratory OR feminist OR "focus group*" OR "grounded theory" OR hermeneutic OR interview OR narrative OR naturalistic OR "participant observation" OR phenomenology OR "qualitative method" OR "qualitative research" OR "qualitative study" OR "thematic analysis" OR "mixed method research" OR "mixed methods research")
#16	#13 OR #14 OR #15
#17	#4 AND #8 AND #12 AND #16

1. Search strategy for Wan Fang	
#1	**主题:**(″痴呆″ OR ″老年痴呆″ OR ″血管性痴呆″ OR ″额颞叶性痴呆″ OR ″帕金森痴呆″ OR ″失智症″ OR ″认知症″ OR ″认知障碍″ OR ″阿尔茨海默″ OR ″阿尔兹海默″ OR ″Alzheimer″ OR ″AD″ OR ″轻度认知障碍″ OR ″MCI″ OR ″路易体痴呆″)
#2	**主题:**(″照护″ OR ″照顾″ OR ″护理″ OR ″管理″ OR ″照料″ OR ″照管″ OR ″照看″ OR ″看护″)
#3	**主题:**(″养老机构″ OR ″养老院″ OR ″护理之家″ OR ″幸福院″ OR ″老人院″ OR ″长期照护机构″ OR ″长期照顾机构″ OR ″长期护理机构″ OR ″长期看护机构″ OR ″敬老院″ OR ″安老院″ OR ″护理院″ OR ″老年人公寓″)
#4	**主题:**(″质性研究″ OR ″定性研究″ OR ″描述性研究″ OR ″民族志″ OR ″现象学″ OR ″扎根理论″ OR ″内容分析″ OR ″主题分析″ OR ″焦点小组″ OR ″解释学″ OR ″访谈″ OR ″叙述″ OR ″混合性研究″)
#5	#1 AND #2 AND #3 AND #4
2. Search strategy for CNKI	
#1	**SU %=** ’痴呆’ + ’老年痴呆’ + ’血管性痴呆’ + ’额颞叶性痴呆’ + ’帕金森痴呆’ + ’失智症’ + ’认知症’ + ’认知障碍’ + ’阿尔茨海默’ + ’阿尔兹海默’ + ’Alzheimer’ + ’AD’ + ’轻度认知障碍’ + ’MCI’ + ’路易体痴呆’
#2	**SU %=** ’照护’ + ’照顾’ + ’护理’ + ’管理’ + ’照料’ + ’照管’ + ’照看’ + ’看护’
#3	**SU %=** ’养老机构’ + ’养老院’ + ’护理之家’ + ’幸福院’ + ’老人院’ + ’长期照护机构’ + ’长期照顾机构’ + ’长期护理机构’ + ’长期看护机构’ + ’敬老院’ + ’安老院’ + ’护理院’ + ’老年人公寓’
#4	**SU %=** ’质性研究’ + ’定性研究’ + ’描述性研究’ + ’民族志’ + ’现象学’ + ’扎根理论’ + ’内容分析’ + ’主题分析’ + ’焦点小组’ + ’解释学’ + ’访谈’ + ’叙述’ + ’混合型研究’
#5	#1 AND #2 AND #3 AND #4
3. Search strategy for CBM	
#1	**″痴呆，血管性″[不加权:扩展] OR ″阿尔茨海默病″[不加权:扩展] OR ″Lewy 体病″[不加权:扩展] OR ″阿尔茨海默病″[不加权:扩展] OR ″认知障碍″[不加权:扩展]**
#2	″痴呆″**[摘要:智能]** OR ″老年痴呆″[摘要:智能] OR ″血管性痴呆″[摘要:智能] OR ″额颞性痴呆″[摘要:智能] OR ″帕金森痴呆″[摘要:智能] OR ″失智症″[摘要:智能] OR ″认知症″[摘要:智能] OR ″认知障碍″[摘要:智能] OR ″阿尔茨海默″[摘要:智能] OR ″阿尔兹海默″[摘要:智能] OR ″Alzheimer″[摘要:智能] OR ″AD″[摘要:智能] OR ″轻度认知障碍″[摘要:智能] OR ″MCI″[摘要:智能] OR ″路易体痴呆″[摘要:智能]
#3	″痴呆″**[标题:智能]** OR ″老年痴呆″[标题:智能] OR ″血管性痴呆″[标题:智能] OR ″额颞性痴呆″[标题:智能] OR ″帕金森痴呆″[标题:智能] OR ″失智症″[标题:智能] OR ″认知症″[标题:智能] OR ″认知障碍″[标题:智能] OR ″阿尔茨海默″[标题:智能] OR ″阿尔兹海默″[标题:智能] OR ″Alzheimer″[标题:智能] OR ″AD″[标题:智能] OR ″轻度认知障碍″[标题:智能] OR ″MCI″[标题:智能] OR ″路易体痴呆″[标题:智能]
#4	#1 OR #2 OR #3

#5	**"护理"[不加权:扩展]**
#6	"照护"**[摘要:智能]** OR "照顾"[摘要:智能] OR "护理"[摘要:智能] OR "管理"[摘要:智能] OR "照料"[摘要:智能] OR "照管"[摘要:智能] OR "照看"[摘要:智能] OR "看护"[摘要:智能]
#7	"照护"**[标题:智能]** OR "照顾"[标题:智能] OR "护理"[标题:智能] OR "管理"[标题:智能] OR "照料"[标题:智能] OR "照管"[标题:智能] OR "照看"[标题:智能] OR "看护"[标题:智能]
#8	#5 OR #6 OR #7
#9	**"老人疗养院"[不加权:扩展]**
#10	"养老机构"**[摘要:智能]** OR "养老院"[摘要:智能] OR "护理之家"[摘要:智能] OR "幸福院"[摘要:智能] OR "老人院"[摘要:智能] OR "长期照护机构"[摘要:智能] OR "长期护理机构"[摘要:智能] OR "长期照顾机构"[摘要:智能] OR "长期看护机构"[摘要:智能] OR "敬老院"[摘要:智能] OR "安老院"[摘要:智能] OR "护理院"[摘要:智能] OR "老年人公寓"[摘要:智能]
#11	"养老机构"**[标题:智能]** OR "养老院"[标题:智能] OR "护理之家"[标题:智能] OR "幸福院"[标题:智能] OR "老人院"[标题:智能] OR "长期照护机构"[标题:智能] OR "长期护理机构"[标题:智能] OR "长期照顾机构"[标题:智能] OR "长期看护机构"[标题:智能] OR "敬老院"[标题:智能] OR "安老院"[标题:智能] OR "护理院"[标题:智能] OR "老年人公寓"[标题:智能]
#12	#9 OR #10 OR #11
#13	"质性研究"**[摘要:智能]** OR "定性研究"[摘要:智能] OR "描述性研究"[摘要:智能] OR "民族志"[摘要:智能] OR "现象学"[摘要:智能] OR "扎根理论"[摘要:智能] OR "内容分析"[摘要:智能] OR "主题分析"[摘要:智能] OR "焦点小组"[摘要:智能] OR "解释学"[摘要:智能] OR "访谈"[摘要:智能] OR "叙述"[摘要:智能] OR "混合型研究"[摘要:智能]
#14	"质性研究"**[标题:智能]** OR "定性研究"[标题:智能] OR "描述性研究"[标题:智能] OR "民族志"[标题:智能] OR "现象学"[标题:智能] OR "扎根理论"[标题:智能] OR "内容分析"[标题:智能] OR "主题分析"[标题:智能] OR "焦点小组"[标题:智能] OR "解释学"[标题:智能] OR "访谈"[标题:智能] OR "叙述"[标题:智能] OR "混合型研究"[标题:智能]
#15	#13 OR #14
#16	#4 AND #8 AND #12 AND #15
4. Search strategy for VIP	
#1	**M**=("痴呆" + "老年痴呆" + "失智症" + "认知症" + "认知障碍" + "阿尔茨海默" + "阿尔兹海默" + "AD" + "轻度认知障碍" + "MCI" + "额颞叶性痴" + "血管性痴呆" + "帕金森痴呆" + "Alzheimer" + "路易体痴呆") OR **R**=("痴呆" + "老年痴呆" + "失智症" + "认知症" + "认知障碍" + "阿尔茨海默" + "阿尔兹海默" + "AD" + "轻度认知障碍" + "MCI" + "额颞叶性痴" + "血管性痴呆" + "帕金森痴呆" + "Alzheimer" + "路易体痴呆")
#2	**M**=("照护" + "护理" + "照顾" + "照料" + "照看" + "照管" + "看护") OR **R**=("照护" + "护理" + "照顾" + "照料" + "照看" + "照管" + "看护")
#3	**M**=("养老机构" + "养老院" + "护理之家" + "幸福院" + "老人院" + "敬老院" + "安老院" + "护理院" + "老年人公寓") OR **R**=("养老机构" + "养老院" + "护理之家" + "幸福院" + "老人院" + "敬老院" + "安老院" + "护理院" + "老年人

	公寓″)
#4	**M=**(″质性研究″ + ″定性研究″ + ″描述性研究″ + ″民族志″ + ″现象学″ + ″扎根理论″ + ″内容分析″ + ″主题分析″ + ″焦点小组″ + ″解释学″ + ″访谈″ + ″叙述″ + ″混合性研究″) OR **R=**(″质性研究″ + ″定性研究″ + ″描述性研究″ + ″民族志″ + ″现象学″ + ″扎根理论″ + ″内容分析″ + ″主题分析″ + ″焦点小组″ + ″解释学″ + ″访谈″ + ″叙述″ + ″混合性研究″)
#5	#1 AND #2 AND #3 AND #4
