# Supplementary material for: Barriers and facilitators to dementia care in long-term care facilities: protocol for a qualitative systematic review and meta-synthesis
Source: BMJ Open. 2023 Nov 1;13(11):e076058. doi: 10.1136/bmjopen-2023-076058 (PMC10626821; doi:10.1136/bmjopen-2023-076058)
Supplement: Supplementary data [file bmjopen-2023-076058supp002.pdf]

JBI Critical Appraisal Checklist for Qualitative Research

Reviewer___________________________________Date__________________________

Author ___________________________________Year________Record Number______

	Yes	No	Unclear	Not applicable
1. Is there congruity between the stated philosophical perspective and the research methodology?	☐	☐	☐	☐
2. Is there congruity between the research methodology and the research question or objectives?	☐	☐	☐	☐
3. Is there congruity between the research methodology and the methods used to collect data?	☐	☐	☐	☐
4. Is there congruity between the research methodology and the representation and analysis of data?	☐	☐	☐	☐
5. Is there congruity between the research methodology and the interpretation of results?	☐	☐	☐	☐
6. Is there a statement locating the researcher culturally or theoretically?	☐	☐	☐	☐
7. Is the influence of the researcher on the research, and vice- versa, addressed?	☐	☐	☐	☐
8. Are participants, and their voices, adequately represented?	☐	☐	☐	☐
9. Is the research ethical according to current criteria or, for recent studies, and is there evidence of ethical approval by an appropriate body?	☐	☐	☐	☐
10. Do the conclusions drawn in the research report flow from the analysis, or interpretation, of the data?	☐	☐	☐	☐

Overall appraisal: Include ☐ Exclude ☐ Seek further info ☐

Comments (Including reason for exclusion)
