# Supplementary material for: Barriers and facilitators to dementia care in long-term care facilities: protocol for a qualitative systematic review and meta-synthesis
Source: BMJ Open. 2023 Nov 1;13(11):e076058. doi: 10.1136/bmjopen-2023-076058 (PMC10626821; doi:10.1136/bmjopen-2023-076058)
Supplement: Supplementary data [file bmjopen-2023-076058supp003.pdf]

JBI QARI Data Extraction Tool for Qualitative Research

Reviewer ______________________ Date ______________________

Author ______________________ Year ______________________

Journal ______________________ Record Number ______________________

Study Description

Methodology

____

Method

____

Phenomena of interest

____

Setting

____

Geographical

____

Cultural

____

Participants

____

Data analysis

____

Authors conclusions

____

Comments

____

Complete Yes ☐ No ☐

Copyright © The Jo0anna Briggs Institute 2014

Findings	Illustration form Publication (page number)	Evidence		
		Unequivocal	Credible	Unsupported

Extraction of findings complete Yes ☐ No ☐

Copyright © The Jo0anna Briggs Institute 2014
