# Supplementary material for: Barriers and facilitators to dementia care in long-term care facilities: protocol for a qualitative systematic review and meta-synthesis
Source: BMJ Open. 2023 Nov 1;13(11):e076058. doi: 10.1136/bmjopen-2023-076058 (PMC10626821; doi:10.1136/bmjopen-2023-076058)
Supplement: Author's manuscript [file bmjopen-2023-076058.draft_revisions.pdf]

BMJ Open is committed to open peer review. As part of this commitment we make the peer review history of every article we publish publicly available.

When an article is published we post the peer reviewers' comments and the authors' responses online. We also post the versions of the paper that were used during peer review. These are the versions that the peer review comments apply to.

The versions of the paper that follow are the versions that were submitted during the peer review process. They are not the versions of record or the final published versions. They should not be cited or distributed as the published version of this manuscript.

BMJ Open is an open access journal and the full, final, typeset and author-corrected version of record of the manuscript is available on our site with no access controls, subscription charges or pay-per-view fees (http://bmjopen.bmj.com).

If you have any questions on BMJ Open's open peer review process please email info.bmjopen@bmj.com

BMJ Open

Barriers and facilitators to dementia care in long-term care facilities：A systematic review and meta-synthesis protocol

Journal:	*BMJ Open*
Manuscript ID	bmjopen-2023-076058
Article Type:	Protocol
Date Submitted by the Author:	26-May-2023

Complete List of Authors:	Zhang, Xi; Xi'an Jiaotong University<br>Guan, Chengguo; Xi'an Jiaotong University<br>He, Jinjie; Xi'an Jiaotong University<br>Wang, Jing; Xi'an Jiaotong University

Keywords:	Systematic Review, Dementia < NEUROLOGY, QUALITATIVE RESEARCH, Protocols & guidelines < HEALTH SERVICES ADMINISTRATION & MANAGEMENT

SCHOLARONE™
Manuscripts

Barriers and facilitators to dementia care in long-term care facilities：A systematic review and meta-synthesis protocol

Authors and affiliation

Xi Zhang[1], Chengguo Guan[1], Jinjie He[1], Jing Wang[1]*

[1]Health Science Center, Xi'an Jiaotong University, Xi'an, Shaanxi, China

Email

Xi Zhang: zhangxi1234560814@163.com

Chengguo Guan: novogcg@stu.xjtu.edu.cn

Jinjie He: hjj521209@stu.xjtu.edu.cn

Jing Wang*: novowj@xjtu.edu.cn

***Corresponding author**

Jing Wang

Health Science Center, Xi'an Jiaotong University

No.76 Yanta West Road, Xi'an, Shaanxi, China

Phone: +86-18789400330

Email: novowj@xjtu.edu.cn

Keywords:

dementia, long-term care facilities, qualitative study, systematic review, protocol

Word count

2012

For peer review only

ABSTRACT

Introduction: Long-term care needs for people with dementia are predicted to increase due to increased life expectancy and dementia diagnoses. Most published meta-integrations of dementia care focus on hospital or home settings, and dementia care in long-term care institutions focuses on a single outcome, such as feeding, behavioral symptoms management, palliative care, and others, which is limited. Integrating these factors is essential to inform the design of better implementation strategies. The present study aims to synthesise qualitative data and examine barriers and facilitators to caring for dementia in long-term care facilities.

Methods and analysis: The present systematic review and meta-synthesis will follow the Joanna Briggs Institute (JBI) guidance for systematic reviews of qualitative evidence. Nine databases (five English and four Chinese) were searched, including Embase, Web of Science, Medline, CINAHL, PsycINFO and Wan Fang Data, China National Knowledge Infrastructure (CNKI), VIP, and Chinese Biomedical Medicine (CBM), from inception to May 2022. Qualitative and mixed-approach researches about barriers and facilitators to caring for dementia in long-term care facilities, which are reported in English or Chinese, will be included. Covidence software will help with study selection, assessment, and data extraction. The JBI Critical Appraisal Checklist for Qualitative Research (2020) will be used for included studies' quality assessment. Data extraction will be based on the JBI-QARI Data Extraction Tool for Qualitative Research. The JBI aggregation approach will be used to synthesises data. We will use the JBI ConQual tool to assess the credibility and dependability of each synthesized finding to establish confidence in the synthesized findings. All review steps will be managed by two reviewers independently, disparities will be discussed. If consensus cannot reach a resolution, a third reviewer will be consulted.

Ethics and dissemination: The present study is a secondary analysis of published qualitative data. So ethical approval is not required.

Registration details: The protocol was registered with the International Prospective Register of Systematic Reviews (PROSPERO) in May 2022, and the registration

For peer review only

number is CRD42022326178.

Keywords: dementia, long-term care facilities, qualitative study, systematic review, protocol

ARTICLE SUMMARY

Strengths and limitations of this study

- The first systematic review and qualitative meta-synthesis to synthesize qualitative evidence that explores the barriers and facilitators to caring for people with dementia in long-term care facilities.
- To achieve a high level of rigor and reliability, this study will be guided by the Joanna Briggs Institute methodology for qualitative systematic reviews. Moreover, the consensus-based analysis will guarantee shared responsibility for interpretive judgments, boosting the accuracy and reliability of results.
- A limitation is the exclusion of studies not written in the English or Chinese language and of grey literature.

INTRODUCTION

Dementia is a degenerative syndrome affecting individuals' cognitive functions. It impacts memory, behavior, thinking, and social abilities as the disease advances. Those affected are about to become increasingly dependent on care from others for all activities of daily living.[1] Previous studies show that age is a critical factor in dementia, and there is no cure for dementia currently.[2] The demographic shift to an aging population has rapidly increased the number of people with dementia. According to the most recent World Health Organization (WHO) figures, 55.2 million people worldwide were living with dementia in 2019, and the number is expected to rise to 139 million in 2050.[3]

Caring for people with dementia requires round-the-clock care, and caregivers must manage behavioral and psychological symptoms (such as aggression, wandering, and depression), placing an overwhelming burden on the caregivers' well-being, especially

For peer review only

in the severe stage of dementia.[4 5] Long-term care facilities are the alternative place when people with dementia cannot get sufficient support at home.[6 7] Long-term care facilities may have different names in different countries, like nursing homes, skilled nursing facilities, assisted living facilities, and residential facilities, providing services to people who cannot live independently.[8] Approximately over 30% of long-term care facility residents have a form of dementia in higher-income economies. [3] In lower-and middle-income countries, informal carers, mostly family members, provide a significant proportion of dementia care nowadays.[3] However, long-term care needs for people with dementia are predicted to increase due to increased life expectancy and dementia diagnoses.

The WHO Framework for Countries to achieve an integrated continuum of long-term care recognizes critical aspects for an integrated continuum service provision in long-term care facilities and guides countries in evaluating and implementing equitable and sustainable care in long-term care facilities.[9] Knowledge-practice gaps about dementia care in long-term care facilities have been revealed by several prior qualitative studies.[10-13] The support from management or resources available,[14] organizational culture or environment,[11] communication,[10] staffing levels,[12 15] and their attitudes[16]to dementia are thought of as key factors associated with dementia care practices in long-term facilities. Insufficient time, lack of relevant expertise, and communication with people with dementia are commonly cited as obstacles. The behavioral and psychological symptoms impact care provision as well, and how to manage these symptoms is still an endeavor[17]. In recent years, qualitative studies are proliferating in nursing research because they effectively complement quantitative research results.[18] Qualitative studies explore detailed explanations and meanings shown by participants, providing an alternative perspective to a more positivist approach.[19] The translation of evidence and clinical citation of qualitative study results have attracted the attention of researchers. Meta-synthesis of qualitative studies can yield broader results because they synthesize a more comprehensive range of participants and descriptions. It integrates the body of knowledge about a topic and, as a result of this process, can produce new interpretations of the phenomenon that contribute to understanding the topic.[20] At

For peer review only

present date, most published meta-synthesis of dementia care focus on hospital[21-23] or home settings[24-26], and most reviews about dementia care in long-term care institutions have a focus on a single outcome, such as feeding,[27] behavioral symptoms management,[28 29] palliative care,[30] and others, which is limited. Integrating these factors is essential to inform the design of better implementation strategies.

Systematic evaluations of qualitative research about dementia care in long-term care facilities are scarce. Therefore, the present study aims to systemically review and synthesise the best available qualitative evidence on barriers and facilitators to caring for people with dementia in long-term care facilities, providing more comprehensive evidence for better service development for dementia care in long-term care facilities.

METHOD AND ANALYSIS

Design

The protocol was registered with the International Prospective Register of Systematic Reviews (PROSPERO) in May 2022, and the registration number is CRD42022326178. This protocol was designed following the Preferred reporting items for systematic review and meta-analysis protocols (PRISMA-P) 2015 statement [31 32] and the Joanna Briggs Institute guidance for systematic reviews of qualitative evidence.[33] The study will run from June 2022 to March 2024.

Eligibility criteria

Type of participants: Studies focus on health professionals (aged≥18) caring for people with dementia in long-term care facilities, including doctors, nurses, and other health professionals. Studies reporting on people with dementia or family member caregivers will be excluded. Studies covering care for multiple disease types (not only care for dementia) will also be excluded.

Phenomena of interest: Studies focus on enablers and barriers to caring for people with dementia in long-term care facilities.

Context: Studies focus on Long-term care facilities,[8] such as nursing homes, skilled nursing facilities, assisted living facilities, nursing institutes, and residential facilities,

For peer review only

providing services to people who cannot live independently. Studies conducted in adult daycare facilities, respite care, and other transitional care facilities will also be included. Types of studies: Qualitative and mixed methods studies with an analytical part of the qualitative content published in English or Chinese will be included. Only the qualitative component of the mixed methods studies will be considered. Quantitative studies, pilot studies, reviews, case reports, case series, reanalysis of data, conference abstracts, and expert opinions will be excluded.

Information sources

Nine databases will be searched, including five English databases and four Chinese databases with no time limit applied: The five English databases are Embase, Web of Science, Medline, CINAHL, PsycINFO and the four Chinese databases are Wan Fang Data, China National Knowledge Infrastructure (CNKI), VIP and Chinese Biomedical Medicine (CBM). References and citations of the included studies will also be searched to identify if there are any other relevant sources.

Search strategy

Keywords were first formed based on the eligibility criteria, and synonyms of each keyword were then analysed to create a logic grid to capture relevant studies. Boolean operators will be used to combine all the keywords. Pilot searches will be conducted to confirm the sensitivity and specificity of the strategy in all databases before the formal search is conducted. The final strategy will be consulted by an expert librarian and reviewed by all team members. The index terms and the search strategy were developed in Web of Science among five English databases and China National Knowledge Infrastructure among four Chinese databases. The Web of Science English search strategy and China National Knowledge Infrastructure Chinese search strategy will be then translated and used for additional databases. We will include studies retrievable from the establishment of the database to May 2022. Search strategies are available in Supplement 1.

Study selection

All search records will be imported to Endnote X9 (V.9.3.3) for duplicates removal and to Covidence software (https://app.covidence.org/reviews/active) for study selection.

For peer review only

Covidence is a systematic review production tool developed by researchers specialized in the systematic review process to standardize the process of conducting systematic reviews. According to the proposed eligibility criteria, title and abstract screening will be conducted by two reviewers (CG and XZ) independently on all records. Then, the full-text review of included studies will be screened in the same way, and the reason for exclusion will be selected and recorded in Covidence. Disparities will be discussed during the screening process via group discussion. If a resolution cannot be reached by consensus, the third and fourth reviewers (JH and JW) will be consulted. Regular group meetings with all the reviewers will be held regularly to discuss and resolve any disagreements. The reference list of each included study will be screened manually by reviewers to determine if there are any citations that meet the eligibility. The results of the screening will be recorded in a PRISMA flow chart.

Risk of bias assessment

The JBI Critical Appraisal Checklist for Qualitative Research (2020) (Supplement 2) will be used for assessing the quality of included studies, which includes ten items that assess the studies' philosophical foundation, methodology, method of data collection, method of data analysis, results interpretation, the logic of studies and research ethics. The four responses "yes," "no," "unclear," and "not applicable" will be used to evaluate each item. Studies would be given a weak grade if fewer than six of the items were answered "yes," a medium rating of 7-8, and a strong rating of 9–10. Only studies having at least a medium ranking will be included. Two reviewers (CG and XZ) will appraise all included studies' methodological quality independently. Disparities will be discussed during the assessment process via discussion. The third and fourth reviewers (JH and JW) will be consulted when a resolution cannot be reached by consensus.

Data extraction

Data extraction will be conducted based on the JBI Qualitative Assessment and Review Instrument (JBI-QARI) Data Extraction Tool for Qualitative Research (Supplement 3) by two reviewers (CG and XZ) independently.[33] According to the tool, the characteristics of included studies and findings with illustrations will be extracted. Information will be extracted using Covidence, including (1) author, publication, year,

For peer review only

country, geographical location, and study setting; (2) participants' characteristics and sample size; (3) research design, methodology, and methods; (4) phenomena of interest and main findings. Prior to the commencement of the data extraction, data of two included studies will be extracted using the data extraction tool to pilot test the tool and minimize the risk of error. According to the JBI review manual, all the findings extracted from the selected studies will be evaluated and divided into three levels based on the degree of support between the finding and its illustration: unequivocal, credible and unsupported.[33] Only the findings rated as unequivocal or credible will be included for data synthesis. Any disagreements between the two reviewers will be discussed and resolved by regular team meetings with the four reviewers.

Data synthesis

The JBI meta aggregation approach with a three-step process will be used for data synthesis after data extraction.[34] In the three-step data synthesis process, the findings will first be extracted from the original studies, then the included findings will be grouped into categories based on similarity of meaning, and similar categories will ultimately be further synthesized into different synthesized findings based on their relationships and relevance to the research objective of this review. This approach focuses on the essence of qualitative research and emphasizes the value and role of qualitative research in an evidence-based healthcare service system, collecting all included studies' findings and summarizing them according to their meanings to make them more targeted, convincing, and generalizing. On the premise of understanding the philosophical foundation and methodology of various qualitative studies, reviewers repeatedly read, understand, analyze, and interpret the meaning of each included findings, combine similar findings, form a new category, and finally summarize the category as synthesized findings. In the present review, the findings of each included study will be read carefully to understand completely before data synthesis. Regular meetings will be held with the four reviewers to reach an agreement on the final synthesized findings, and any conflicting opinions between the reviewers will be settled by thorough discussions.

Assessing the certainty of findings

For peer review only

We will use the JBI ConQual tool to assess the dependability and credibility of each synthesised finding to establish confidence in the synthesized findings.[35] Dependability mainly examines the quality of the original studies included. Reliability primarily examines the consistency between synthesised findings and the supporting data. First, meta-synthesis findings will be initially assumed to be graded as high, and then the quality of evidence will be downgraded based on the results of stability and credibility assessments, and finally each synthesized finding will be rated high, moderate, low, or very low.[35]

Patient and public involvement

None.

Ethics and dissemination

The present study is a secondary analysis of published qualitative data; there is no concern for privacy. So ethical approval is not required for the present study. However, the research ethics of each included original study will be assessed rigorously. The findings may be disseminated through peer-reviewed publications, conference papers or elsewhere.

DISCUSSION

The expected increase in the demand for high-quality dementia care in long-term care facilities warrants an evaluation of the facilitators and barriers to improving dementia care in these settings. The present study will synthesise the best available qualitative evidence in this area, and the results of this study will include: the thematic meta-synthesis results of both barriers and facilitators to caring for people with dementia in long-term care facilities.

Acknowledgments

We gratitude for the help and support of the National Social Science Fund of China.

Author Contributions

JW and CG conceived the topic of this study. All team members (JW, CG, JH, XZ) participated in the discussion of the study design and made significant comments. JW

For peer review only

and CG constructed the search strategy. CG, JH, XZ, and JW will conduct data selection, assessment, extraction, and synthesis. XZ drafted the protocol manuscript, and then the manuscript was reviewed by JW. All authors approved the submitted protocol and are accountable for its content.

Funding statement

This work was supported by The National Social Science Fund of China (Grant No. 20XRK004).

Competing interests

none

For peer review only

References

1. World Alzheimer Report 2021: Journey through the diagnosis of dementia. *Alzheimer's Disease International,* 2021. https://www.alzint.org/resource/world-alzheimer-report-2021/.

2. Jia L, Du Y, Chu L, et al. Prevalence, risk factors, and management of dementia and mild cognitive impairment in adults aged 60 years or older in China: a cross-sectional study. *Lancet Public Health* 2020;5(12):e661-e71. doi: 10.1016/s2468-2667(20)30185-7

3. WHO Global status report on the public health response to dementia Executive summary. *World Health Organization (WHO),* 2021. https://www.who.int/multi-media/details/global-status-report-on-the-public-health-response-to-dementia.

4. Wang J, Xiao LD, Li X, et al. Caregiver distress and associated factors in dementia care in the community setting in China. *Geriatr Nurs* 2015;36(5):348-54. doi: 10.1016/j.gerinurse.2015.04.013

5. Connors MH, Seeher K, Teixeira-Pinto A, et al. Dementia and caregiver burden: A three-year longitudinal study. *Int J Geriatr Psychiatry* 2020;35(2):250-58. doi: 10.1002/gps.5244

6. Xiao LD, Wang J, He GP, et al. Family caregiver challenges in dementia care in Australia and China: a critical perspective. *BMC Geriatr* 2014;14:6. doi: 10.1186/1471-2318-14-6

7. Gaugler JE, Mittelman MS, Hepburn K, et al. Predictors of change in caregiver burden and depressive symptoms following nursing home admission. *Psychol Aging* 2009;24(2):385-96. doi: 10.1037/a0016052

8. Preventing and managing COVID-19 across long-term care services: Policy brief, 24 July 2020. *world Health Organization (WHO),* 2020. https://www.who.int/publications/i/item/WHO-2019-nCoV-Policy_Brief-Long-term_Care-2020.1.

9. Framework for countries to achieve an integrated continuum of long-term care. *World Health Organization (WHO)* , 2021. https://www.who.int/publications/i/item/9789240038844.

10. Barbosa A, Nolan M, Sousa L, et al. Dementia in Long-term Care Homes: Direct Care Workers' Difficulties. *Procedia - Social and Behavioral Sciences* 2014;140:172-77. org/10.1016/j.sbspro.2014.04.405

11. Zhao Y, Liu L, Ding Y, et al. Understanding dementia care in care home setting in China: An exploratory qualitative study. *Health Soc Care Community* 2021;29(5):1511-21. doi: 10.1111/hsc.13213

12. Kong EH, Kim H, Kim H. Nursing home staff's perceptions of barriers and needs in implementing person-centred care for people living with dementia: A qualitative study. *J Clin Nurs* 2022;31(13-14):1896-906. doi: 10.1111/jocn.15729

13. Law K, Patterson TG, Muers J. Experiences of healthcare assistants working with clients with dementia in residential care homes. *Dementia (London)* 2019;18(2):644-59. doi: 10.1177/1471301216688396

14. Jiang L, Sun F, Bonifas RP, et al. Leadership challenges and strategies to dementia care in Chinese faith-based nursing homes: A qualitative study. *J Nurs Manag* 2022;30(3):777-84. doi: 10.1111/jonm.13564

15. Cohen-Mansfield J, Meschiany G. Israeli nursing home staff perspectives on challenges to quality care for residents with dementia. *Geriatr Nurs* 2022;44:15-23. doi: 10.1016/j.gerinurse.2021.12.012

16. Gerritsen DL, van Beek APA, Woods RT. Relationship of care staff attitudes with social well-being and challenging behavior of nursing home residents with dementia: a

For peer review only

cross sectional study. *Aging Ment Health* 2019;23(11):1517-23. doi: 10.1080/13607863.2018.1506737

17. O'Donnell E, Holland C, Swarbrick C. Strategies used by care home staff to manage behaviour that challenges in dementia: A systematic review of qualitative studies. *Int J Nurs Stud* 2022;133:104260. doi: 10.1016/j.ijnurstu.2022.104260

18. Wang J, Li X, Liu W, et al. The positive aspects of caregiving in dementia: A scoping review and bibliometric analysis. *Front Public Health* 2022;10:985391. doi: 10.3389/fpubh.2022.985391

19. Lindgren BM, Lundman B, Graneheim UH. Abstraction and interpretation during the qualitative content analysis process. *Int J Nurs Stud* 2020;108:103632. doi: 10.1016/j.ijnurstu.2020.103632

20. Evans D, Pearson A. Systematic reviews of qualitative research. *Clinical Effectiveness in Nursing* 2005;5:111-19. doi: 10.1054/cein.2001.0219

21. Brossard Saxell T, Ingvert M, Lethin C. Facilitators for person-centred care of inpatients with dementia: A meta-synthesis of registered nurses' experiences. *Dementia (London)* 2021;20(1):188-212. doi: 10.1177/1471301219871408

22. Moonga J, Likupe G. A systematic literature review on nurses' and health care support workers' experiences of caring for people with dementia on orthopaedic wards. *J Clin Nurs* 2016;25(13-14):1789-804. doi: 10.1111/jocn.13158

23. Digby R, Lee S, Williams A. The experience of people with dementia and nurses in hospital: an integrative review. *J Clin Nurs* 2017;26(9-10):1152-71. doi: 10.1111/jocn.13429

24. Köhler K, Dreyer J, Hochgraeber I, et al. Towards a middle-range theory of 'Stability of home-based care arrangements for people living with dementia' (SoCA-Dem): findings from a meta-study on mixed research. *BMJ Open* 2021;11(4):e042515. doi: 10.1136/bmjopen-2020-042515

25. Dawson A, Bowes A, Kelly F, et al. Evidence of what works to support and sustain care at home for people with dementia: a literature review with a systematic approach. *BMC Geriatr* 2015;15:59. doi: 10.1186/s12877-015-0053-9

26. Marulappa N, Anderson NN, Bethell J, et al. How to implement person-centred care

For peer review only

and support for dementia in outpatient and home/community settings: Scoping review. *BMC Health Serv Res* 2022;22(1):541. doi: 10.1186/s12913-022-07875-w

27. Faraday J, Salis C, Barrett A. Equipping Nurses and Care Staff to Manage Mealtime Difficulties in People With Dementia: A Systematic Scoping Review of Training Needs and Interventions. *Am J Speech Lang Pathol* 2019;28(2):717-42. doi: 10.1044/2018_ajslp-18-0062

28. Walsh KA, Dennehy R, Sinnott C, et al. Influences on Decision-Making Regarding Antipsychotic Prescribing in Nursing Home Residents With Dementia: A Systematic Review and Synthesis of Qualitative Evidence. *J Am Med Dir Assoc* 2017;18(10):897.e1-97.e12. doi: 10.1016/j.jamda.2017.06.032

29. Jennings AA, Foley T, Walsh KA, et al. General practitioners' knowledge, attitudes, and experiences of managing behavioural and psychological symptoms of dementia: A mixed-methods systematic review. *Int J Geriatr Psychiatry* 2018;33(9):1163-76. doi: 10.1002/gps.4918

30. van der Steen JT, Radbruch L, Hertogh CM, et al. White paper defining optimal palliative care in older people with dementia: a Delphi study and recommendations from the European Association for Palliative Care. *Palliat Med* 2014;28(3):197-209. doi: 10.1177/0269216313493685

31. Moher D, Shamseer L, Clarke M, et al. Preferred reporting items for systematic review and meta-analysis protocols (PRISMA-P) 2015 statement. *Syst Rev* 2015;4(1):1. doi: 10.1186/2046-4053-4-1

32. Shamseer L, Moher D, Clarke M, et al. Preferred reporting items for systematic review and meta-analysis protocols (PRISMA-P) 2015: elaboration and explanation. *Bmj* 2015;350:g7647. doi: 10.1136/bmj.g7647

33. Lockwood C, Porritt K, Munn Z, et al. Chapter 2: Systematic reviews of qualitative evidence. *JBI Manual for Evidence Synthesis*; JBI, 2020. https://synthesismanual.jbi.global.

34. Lockwood C, Munn Z, Porritt K. Qualitative research synthesis: methodological guidance for systematic reviewers utilizing meta-aggregation. *Int J Evid Based Healthc* 2015;13(3):179-87. doi: 10.1097/xeb.0000000000000062

For peer review only

35. Munn Z, Porritt K, Lockwood C, et al. Establishing confidence in the output of qualitative research synthesis: the ConQual approach. *BMC Med Res Methodol* 2014;14:108. doi: 10.1186/1471-2288-14-108

For peer review only

Barriers and facilitators to dementia care in long-term care facilities：A systematic review and meta-synthesis protocol

Supplement file 1 - Searched databases and search strategies

1. Search strategy for Web of Science	
#1	TI=(dement* OR "presenile dementia" OR "cognitive impairment" OR "cognitive dysfunction" OR "Alzheimer's disease" OR "Alzheimer's dementia" OR AD OR "mild cognitive impairment" OR "huntington disease" OR "lewy body" OR "chronic cerebrovascular" OR "parkinson disease dementia" OR "parkinson dementia" OR “lewy bodies” OR “lewy neurites” OR “dementia, vascular” OR “vascular dementia*”) OR AB=(dementia OR "presenile dementia" OR "cognitive impairment" OR "cognitive dysfunction" OR "Alzheimer's disease" OR "Alzheimer's dementia" OR AD OR "mild cognitive impairment" OR "huntington disease" OR "lewy body" OR "chronic cerebrovascular" OR "parkinson disease dementia" OR "parkinson dementia" OR “cognitive dysfunction” OR “lewy bodies” OR “lewy neurites” OR “dementia, vascular” OR “vascular dementia*”)
#2	TI=(nursing OR "practical nursing" OR "care, nursing" OR "management, nursing Care" OR "care, long-term" OR "long term care" OR care OR caring OR caregiving OR manag* OR "long-term care") OR AB=(nursing OR "practical nursing" OR "care, nursing" OR "management, nursing Care" OR "care, long-term" OR "long term care" OR care OR caring OR caregiving OR manag* OR "long-term care")
#3	TI=("elderly care institution" OR "nursing home*" OR "health care setting" OR "long term care facilities" OR "residential aged care" OR "care home*" OR "nursing home*" OR "residential facilities" OR "homes for aged" OR "residential home*" OR "residential facilit*" OR "nursing home facility*" OR "nursing home care" OR "nursing home placement" OR "old people's home*" OR "homes for the aged" OR "old age home" OR "old age homes" OR "housing for the elderly") OR AB=("elderly care institution" OR "nursing home*" OR "health care setting" OR "long term care facilities" OR "residential aged care" OR "care home*" OR "nursing home*" OR "residential facilities" OR "homes for aged" OR "residential home*" OR "residential facilit*" OR "nursing home facility*" OR "nursing home care" OR "nursing home placement" OR "old people's home*" OR "homes for the aged" OR "old age home" OR "old age homes" OR "housing for the elderly")
#4	TI=("nursing methodology" OR "case study" OR "constant comparison" OR "content analysis" OR "descriptive study" OR "discourse analysis" OR ethnography OR exploratory OR feminist OR "focus group*" OR "grounded theory" OR hermeneutic OR interview OR narrative OR naturalistic OR "participant observation" OR phenomenology OR "qualitative method" OR "qualitative research" OR "qualitative study" OR "thematic analysis" OR "mixed method research" OR "mixed methods research") OR AB=("nursing methodology" OR "case study" OR "constant comparison" OR "content analysis" OR "descriptive study" OR "discourse analysis" OR ethnography OR exploratory OR feminist OR "focus group*" OR "grounded theory" OR hermeneutic OR interview OR narrative OR naturalistic OR "participant observation"

For peer review only

	OR phenomenology OR "qualitative method" OR "qualitative research" OR "qualitative study" OR "thematic analysis" OR "mixed method research" OR "mixed methods research")
#5	#1 AND #2 AND #3 AND #4
2. Search strategy for Embase	
#1	'Alzheimer disease'/exp OR 'dementia'/exp OR 'mild cognitive impairment'/exp OR 'Lewy body'/exp OR 'multiinfarct dementia'/exp
#2	dement*:ti,ab OR 'presenile dementia':ti,ab OR 'cognitive impairment':ti,ab OR alzheimer*:ti,ab OR ad:ti,ab OR 'mild cognitive impairment':ti,ab OR 'huntington disease':ti,ab OR 'lewy body':ab OR 'chronic cerebrovascular':ti,ab OR 'parkinson disease dementia':ti,ab OR 'parkinson dementia':ti,ab OR 'cognitive dysfunction':ti,ab OR 'lewy bodies':ti,ab OR 'lewy neurites':ti,ab OR 'dementia, vascular':ti,ab OR 'multiinfarct dementia':ab,ti
#3	#1 OR #2
#4	'management'/exp OR 'nursing'/exp OR 'practical nursing'/exp OR 'care'/exp OR 'custodial care'/exp OR 'nursing care'/exp OR 'long term care'/exp
#5	nursings:ti,ab OR 'practical nursing':ti,ab OR 'nursing care':ti,ab OR 'management, nursing care':ti,ab OR 'care, long-term':ti,ab OR 'long term care':ti,ab OR care:ti,ab OR caring:ti,ab OR caregiving:ti,ab OR manag*:ti,ab OR 'long-term care':ti,ab
#6	#4 OR #5
#7	'nursing home'/exp OR 'residential home'/exp OR 'home for the aged'/exp
#8	'elderly care institution':ti,ab OR 'health care setting':ti,ab OR 'long term care facilities':ti,ab OR 'residential aged care':ti,ab OR 'care home*':ti,ab OR 'nursing home*':ti,ab OR 'residential facilities':ti,ab OR 'homes for aged':ti,ab OR 'residential home*':ti,ab OR 'residential facilit*':ti,ab OR 'nursing home facility*':ti,ab OR 'nursing home care':ti,ab OR 'nursing home placement':ti,ab OR 'homes for the aged':ti,ab OR 'old age home':ti,ab OR 'old age homes':ti,ab OR 'housing for the elderly':ti,ab
#9	#7 OR #8
#10	'qualitative research'/exp
#11	'nursing methodology':ti,ab OR 'case study':ti,ab OR 'constant comparison':ti,ab OR 'content analysis':ti,ab OR 'descriptive study':ti,ab OR 'discourse analysis':ti,ab OR ethnography:ti,ab OR exploratory:ti,ab OR feminist:ti,ab OR 'focus group*':ti,ab OR 'grounded theory':ti,ab OR hermeneutic:ti,ab OR interview:ti,ab OR narrative:ti,ab OR naturalistic:ti,ab OR 'participant observation':ti,ab OR phenomenology:ti,ab OR 'qualitative method':ti,ab OR 'qualitative research':ti,ab OR 'qualitative study':ti,ab OR 'thematic analysis':ti,ab OR 'mixed method research':ti,ab OR 'mixed methods research':ti,ab
#12	#10 OR #11
#13	#3 AND #6 AND #9 AND #12 AND
3. Search strategy for Medline	
#1	exp Dementia/ or exp Frontotemporal Dementia/ or exp Dementia, Vascular/ or Cognitive Dysfunction/ or exp Lewy Bodies/ or exp Parkinson Disease/
#2	(dement* or "presenile dementia" or "cognitive impairment" or "cognitive dysfunction" or "Alzheimer's disease" or "Alzheimer's dementia" or AD or "mild cognitive impairment" or "huntington disease" or "lewy body" or "chronic cerebrovascular" or "parkinson disease

For peer review only

	dementia" or "parkinson dementia" or "parkinson dementia").tw.
#3	#1 or #2
#4	exp Nursing/ or exp Nursing, Practical/ or exp Custodial Care/ or Nursing Care/ or exp Long-Term Care/
#5	(nursing or "practical nursing" or "care, nursing" or "management, nursing Care" or "care, long-term" or "long term care" or care or caring or caregiving or manag* or "long-term care").tw.
#6	#4 or #5
#7	exp Nursing Homes/ or exp Residential Facilities/ or Homes for the Aged/
#8	("elderly care institution" or "nursing home*" or "health care setting" or "long term care facilities" or "residential aged care" or "care home*" or "nursing home*" or "residential facilities" or "homes for aged" or "residential home*" or "residential facilit*" or "nursing home facility*" or "nursing home care" or "nursing home placement" or "old people's home*" or "homes for the aged" or "old age home" or "old age homes" or "housing for the elderly").tw.
#9	#7 or #8
#10	exp Qualitative Research/
#11	("nursing methodology" or "case study" or "constant comparison" or "content analysis" or "descriptive study" or "discourse analysis" or ethnography or exploratory or feminist or "focus group*" or "grounded theory" or hermeneutic or interview or narrative or naturalistic or "participant observation" or phenomenology or "qualitative method" or "qualitative research" or "qualitative study" or "thematic analysis" or "mixed method research" or "mixed methods research").tw.
#12	#10 or #11
#13	#3 and #6 and #9 and #12
4. Search strategy for CINAHL	
#1	(MH "Dementia+") OR (MH "Frontotemporal Dementia+") OR (MH "Dementia, Vascular+") OR (MH "Lewy Body Disease") OR (MH "Mild Cognitive Impairment")
#2	TI (dement* OR "presenile dementia" OR "cognitive impairment" OR "cognitive dysfunction" OR "Alzheimer's disease" OR "Alzheimer's dementia" OR AD OR "mild cognitive impairment" OR "huntington disease" OR "lewy body" OR "chronic cerebrovascular" OR "parkinson disease dementia" OR "parkinson dementia" OR “lewy bodies” OR “lewy neurites” OR “dementia, vascular” OR “vascular dementia*”)
#3	AB (dement* OR "presenile dementia" OR "cognitive impairment" OR "cognitive dysfunction" OR "Alzheimer's disease" OR "Alzheimer's dementia" OR AD OR "mild cognitive impairment" OR "huntington disease" OR "lewy body" OR "chronic cerebrovascular" OR "parkinson disease dementia" OR "parkinson dementia" OR “lewy bodies” OR “lewy neurites” OR “dementia, vascular” OR “vascular dementia*”)
#4	#1 OR #2 OR #3
#5	(MH "Caring+") OR (MH "Long Term Care") OR (MH "Management+")
#6	TI (nursing OR "practical nursing" OR "care, nursing" OR "management, nursing Care" OR "care, long-term" OR "long term care" OR care OR caring OR caregiving OR manag* OR "long-term care")
#7	AB (nursing OR "practical nursing" OR "care, nursing" OR "management, nursing Care" OR

	"care, long-term" OR "long term care" OR care OR caring OR caregiving OR manag* OR "long-term care")
#8	#5 OR #6 OR #7
#9	(MH "Nursing Homes+") OR (MH "Residential Facilities+")
#10	TI ("elderly care institution" OR "nursing home*" OR "health care setting" OR "long term care facilities" OR "residential aged care" OR "care home*" OR "nursing home*" OR "residential facilities" OR "homes for aged" OR "residential home*" OR "residential facilit*" OR "nursing home facility*" OR "nursing home care" OR "nursing home placement" OR "old people's home*" OR "homes for the aged" OR "old age home" OR "old age homes" OR "housing for the elderly")
#11	AB ("elderly care institution" OR "nursing home*" OR "health care setting" OR "long term care facilities" OR "residential aged care" OR "care home*" OR "nursing home*" OR "residential facilities" OR "homes for aged" OR "residential home*" OR "residential facilit*" OR "nursing home facility*" OR "nursing home care" OR "nursing home placement" OR "old people's home*" OR "homes for the aged" OR "old age home" OR "old age homes" OR "housing for the elderly")
#12	#9 OR #10 OR #11
#13	(MH "Qualitative Studies+")
#14	TI ("nursing methodology" OR "case study" OR "constant comparison" OR "content analysis" OR "descriptive study" OR "discourse analysis" OR ethnography OR exploratory OR feminist OR "focus group*" OR "grounded theory" OR hermeneutic OR interview OR narrative OR naturalistic OR "participant observation" OR phenomenology OR "qualitative method" OR "qualitative research" OR "qualitative study" OR "thematic analysis" OR "mixed method research" OR "mixed methods research")
#15	AB ("nursing methodology" OR "case study" OR "constant comparison" OR "content analysis" OR "descriptive study" OR "discourse analysis" OR ethnography OR exploratory OR feminist OR "focus group*" OR "grounded theory" OR hermeneutic OR interview OR narrative OR naturalistic OR "participant observation" OR phenomenology OR "qualitative method" OR "qualitative research" OR "qualitative study" OR "thematic analysis" OR "mixed method research" OR "mixed methods research")
#16	#13 OR #14 OR #15
#17	#4 AND #8 AND #12 AND #16
5. Search strategy for PsycINFO	
#1	(DE "Dementia") OR (DE "Dementia with Lewy Bodies") OR (DE "Vascular Dementia") OR (DE "Alzheimer's Disease") OR (DE "Cognitive Impairment") OR (DE "Mild Cognitive Impairment")
#2	TI (dement* OR "presenile dementia" OR "cognitive impairment" OR "cognitive dysfunction" OR "Alzheimer's disease" OR "Alzheimer's dementia" OR "AD" OR "mild cognitive impairment" OR "huntington disease" OR "lewy body" OR "chronic cerebrovascular" OR "parkinson disease dementia" OR "parkinson dementia" OR “lewy bodies” OR “lewy neurites” OR “dementia, vascular” OR “vascular dementia*”)
#3	AB (dement* OR "presenile dementia" OR "cognitive impairment" OR "cognitive dysfunction" OR "Alzheimer's disease" OR "Alzheimer's dementia" OR "AD" OR "mild cognitive impairment" OR "huntington disease" OR "lewy body" OR "chronic

	cerebrovascular" OR "parkinson disease dementia" OR "parkinson dementia" OR “lewy bodies” OR “lewy neurites” OR “dementia, vascular” OR “vascular dementia*”)
#4	#1 OR #2 OR #3
#5	(DE "Nursing") OR (DE "Caregiving") OR (DE "Long Term Care") OR (DE "Management")
#6	TI (nursing OR "practical nursing" OR "care, nursing" OR "management, nursing Care" OR "care, long-term" OR "long term care" OR care OR caring OR caregiving OR cargiving OR manag* OR "long-term care")
#7	AB (nursing OR "practical nursing" OR "care, nursing" OR "management, nursing Care" OR "care, long-term" OR "long term care" OR care OR caring OR caregiving OR manag* OR "long-term care")
#8	#5 OR #6 OR #7
#9	(DE "Nursing Homes") OR (DE "Residential Care Institutions")
#10	TI ("elderly care institution" OR "nursing home*" OR "health care setting" OR "long term care facilities" OR "residential aged care" OR "care home*" OR "nursing home*" OR "residential facilities" OR "homes for aged" OR "residential home*" OR "residential facilit*" OR "nursing home facility*" OR "nursing home care" OR "nursing home placement" OR "old people's home*" OR "homes for the aged" OR "old age home" OR "old age homes" OR "housing for the elderly")
#11	AB ("elderly care institution" OR "nursing home*" OR "health care setting" OR "long term care facilities" OR "residential aged care" OR "care home*" OR "nursing home*" OR "residential facilities" OR "homes for aged" OR "residential home*" OR "residential facilit*" OR "nursing home facility*" OR "nursing home care" OR "nursing home placement" OR "old people's home*" OR "homes for the aged" OR "old age home" OR "old age homes" OR "housing for the elderly")
#12	#9 OR #10 OR #11
#13	DE "Focus Group" OR DE "Focus Group Interview" OR DE "Qualitative Methods" OR DE "Grounded Theory" OR DE "Interpretative Phenomenological Analysis" OR DE "Narrative Analysis" OR DE "Semi-Structured Interview" OR DE "Thematic Analysis"
#14	TI ("nursing methodology" OR "case study" OR "constant comparison" OR "content analysis" OR "descriptive study" OR "discourse analysis" OR ethnography OR exploratory OR feminist OR "focus group*" OR "grounded theory" OR hermeneutic OR interview OR narrative OR naturalistic OR "participant observation" OR phenomenology OR "qualitative method" OR "qualitative research" OR "qualitative study" OR "thematic analysis" OR "mixed method research" OR "mixed methods research")
#15	AB ("nursing methodology" OR "case study" OR "constant comparison" OR "content analysis" OR "descriptive study" OR "discourse analysis" OR ethnography OR exploratory OR feminist OR "focus group*" OR "grounded theory" OR hermeneutic OR interview OR narrative OR naturalistic OR "participant observation" OR phenomenology OR "qualitative method" OR "qualitative research" OR "qualitative study" OR "thematic analysis" OR "mixed method research" OR "mixed methods research")
#16	#13 OR #14 OR #15
#17	#4 AND #8 AND #12 AND #16

1. Search strategy for Wan Fang	
#1	主题:("痴呆" OR "老年痴呆" OR "血管性痴呆" OR "额颞叶性痴呆" OR "帕金森痴呆" OR "失智症" OR "认知症" OR "认知障碍" OR "阿尔茨海默" OR "阿尔兹海默" OR "Alzheimer" OR "AD" OR "轻度认知障碍" OR "MCI" OR "路易体痴呆")
#2	主题:("照护" OR "照顾" OR "护理" OR "管理" OR "照料" OR "照管" OR "照看" OR "看护")
#3	主题:("养老机构" OR "养老院" OR "护理之家" OR "幸福院" OR "老人院" OR "长期照护机构" OR "长期照顾机构" OR "长期护理机构" OR "长期看护机构" OR "敬老院" OR "安老院" OR "护理院" OR "老年人公寓")
#4	主题:("质性研究" OR "定性研究" OR "描述性研究" OR "民族志" OR "现象学" OR "扎根理论" OR "内容分析" OR "主题分析" OR "焦点小组" OR "解释学" OR "访谈" OR "叙述" OR "混合性研究")
#5	#1 AND #2 AND #3 AND #4
注	主题:(质性研究 OR 定性研究 OR 描述性研究 OR 民族志 OR 现象学 OR 扎根理论 OR 内容分析 OR 主题分析 OR 话语分析 OR 焦点小组 OR 解释学 OR 访谈 OR 叙述 OR 自然主义 OR 参与者观察 OR 混合性研究) AND 主题:(痴呆 OR 老年痴呆 OR 血管性痴呆 OR 额颞叶性痴呆 OR 帕金森痴呆 OR 失智症 OR 认知症 OR 认知障碍 OR 阿尔茨海默 OR 阿尔兹海默 OR "Alzheimer's" OR AD OR 轻度认知障碍 OR MCI OR 路易体痴呆) AND 主题:(照护 OR 照顾 OR 护理 OR 管理 OR 照料 OR 照管 OR 照看) AND 主题:(养老机构 OR 养老院 OR 护理之家 OR 幸福院 OR 老人院 OR 长期照护机构 OR 长期照顾机构 OR 长期护理机构 OR 敬老院 OR 安老院 OR 护理院 OR 老年人公寓)(没有加引号)
2. Search strategy for CNKI	
#1	SU %= ’痴呆’ + ’老年痴呆’ + ’血管性痴呆’ + ’额颞叶性痴呆’ + ’帕金森痴呆’ + ’失智症’ + ’认知症’ + ’认知障碍’ + ’阿尔茨海默’ + ’阿尔兹海默’ + ’Alzheimer’ + ’AD’ + ’轻度认知障碍’ + ’MCI’ + ’路易体痴呆’
#2	SU %= ’照护’ + ’照顾’ + ’护理’ + ’管理’ + ’照料’ + ’照管’ + ’照看’ + ’看护’
#3	SU %= ’养老机构’ + ’养老院’ + ’护理之家’ + ’幸福院’ + ’老人院’ + ’长期照护机构’ + ’长期照顾机构’ + ’长期护理机构’ + ’长期看护机构’ + ’敬老院’ + ’安老院’ + ’护理院’ + ’老年人公寓’
#4	SU %= ’质性研究’ + ’定性研究’ + ’描述性研究’ + ’民族志’ + ’现象学’ + ’扎根理论’ + ’内容分析’ + ’主题分析’ + ’焦点小组’ + ’解释学’ + ’访谈’ + ’叙述’ + ’混合型研究’
#5	#1 AND #2 AND #3 AND #4
3. Search strategy for CBM	
#1	"痴呆，血管性"[不加权:扩展] OR "阿尔茨海默病"[不加权:扩展] OR "Lewy 体病"[不加权:扩展] OR "阿尔茨海默病"[不加权:扩展] OR "认知障碍"[不加权:扩展]
#2	"痴呆"[摘要:智能] OR "老年痴呆"[摘要:智能] OR "血管性痴呆"[摘要:智能]

For peer review only

	OR "额颞性痴呆"[摘要:智能] OR "帕金森痴呆"[摘要:智能] OR "失智症"[摘要:智能] OR "认知症"[摘要:智能] OR "认知障碍"[摘要:智能] OR "阿尔茨海默"[摘要:智能] OR "阿尔兹海默"[摘要:智能] OR "Alzheimer"[摘要:智能] OR "AD"[摘要:智能] OR "轻度认知障碍"[摘要:智能] OR "MCI"[摘要:智能] OR "路易体痴呆"[摘要:智能]
#3	"痴呆"[标题:智能] OR "老年痴呆"[标题:智能] OR "血管性痴呆"[标题:智能] OR "额颞性痴呆"[标题:智能] OR "帕金森痴呆"[标题:智能] OR "失智症"[标题:智能] OR "认知症"[标题:智能] OR "认知障碍"[标题:智能] OR "阿尔茨海默"[标题:智能] OR "阿尔兹海默"[标题:智能] OR "Alzheimer"[标题:智能] OR "AD"[标题:智能] OR "轻度认知障碍"[标题:智能] OR "MCI"[标题:智能] OR "路易体痴呆"[标题:智能]
#4	#1 OR #2 OR #3
#5	"护理"[不加权:扩展]
#6	"照护"[摘要:智能] OR "照顾"[摘要:智能] OR "护理"[摘要:智能] OR "管理"[摘要:智能] OR "照料"[摘要:智能] OR "照管"[摘要:智能] OR "照看"[摘要:智能] OR "看护"[摘要:智能]
#7	"照护"[标题:智能] OR "照顾"[标题:智能] OR "护理"[标题:智能] OR "管理"[标题:智能] OR "照料"[标题:智能] OR "照管"[标题:智能] OR "照看"[标题:智能] OR "看护"[标题:智能]
#8	#5 OR #6 OR #7
#9	"老人疗养院"[不加权:扩展]
#10	"养老机构"[摘要:智能] OR "养老院"[摘要:智能] OR "护理之家"[摘要:智能] OR "幸福院"[摘要:智能] OR "老人院"[摘要:智能] OR "长期照护机构"[摘要:智能] OR "长期护理机构"[摘要:智能] OR "长期照顾机构"[摘要:智能] OR "长期看护机构"[摘要:智能] OR "敬老院"[摘要:智能] OR "安老院"[摘要:智能] OR "护理院"[摘要:智能] OR "老年人公寓"[摘要:智能]
#11	"养老机构"[标题:智能] OR "养老院"[标题:智能] OR "护理之家"[标题:智能] OR "幸福院"[标题:智能] OR "老人院"[标题:智能] OR "长期照护机构"[标题:智能] OR "长期护理机构"[标题:智能] OR "长期照顾机构"[标题:智能] OR "长期看护机构"[标题:智能] OR "敬老院"[标题:智能] OR "安老院"[标题:智能] OR "护理院"[标题:智能] OR "老年人公寓"[标题:智能]
#12	#9 OR #10 OR #11
#13	"质性研究"[摘要:智能] OR "定性研究"[摘要:智能] OR "描述性研究"[摘要:智能] OR "民族志"[摘要:智能] OR "现象学"[摘要:智能] OR "扎根理论"[摘要:智能] OR "内容分析"[摘要:智能] OR "主题分析"[摘要:智能] OR "焦点小组"[摘要:智能] OR "解释学"[摘要:智能] OR "访谈"[摘要:智能] OR "叙述"[摘要:智能] OR "混合型研究"[摘要:智能]
#14	"质性研究"[标题:智能] OR "定性研究"[标题:智能] OR "描述性研究"[标题:智能] OR "民族志"[标题:智能] OR "现象学"[标题:智能] OR "扎根理论"[标题:智能] OR "内容分析"[标题:智能] OR "主题分析"[标题:智能] OR "焦点小组"[标题:智能] OR "解释学"[标题:智能] OR "访谈"[标题:智能] OR "叙述"[标题:智能] OR "混合型研究"[标题:智能]
#15	#13 OR #14
#16	#4 AND #8 AND #12 AND #15

For peer review only

4. Search strategy for VIP	
#1	M=(″痴呆″ + ″老年痴呆″ + ″失智症″ + ″认知症″ + ″认知障碍″ + ″阿尔茨海默″ + ″阿尔兹海默″ + ″AD″ + ″轻度认知障碍″ + ″MCI″ + ″额颞叶性痴″ + ″血管性痴呆″ + ″帕金森痴呆″ + ″Alzheimer″ + ″路易体痴呆″) OR R=(″痴呆″ + ″老年痴呆″ + ″失智症″ + ″认知症″ + ″认知障碍″ + ″阿尔茨海默″ + ″阿尔兹海默″ + ″AD″ + ″轻度认知障碍″ + ″MCI″ + ″额颞叶性痴″ + ″血管性痴呆″ + ″帕金森痴呆″ + ″Alzheimer″ + ″路易体痴呆″)
#2	M=(″照护″ + ″护理″ + ″照顾″ + ″照料″ + ″照看″ + ″照管″ + ″看护″) OR R=(″照护″ + ″护理″ + ″照顾″ + ″照料″ + ″照看″ + ″照管″ + ″看护″)
#3	M=(″养老机构″ + ″养老院″ + ″护理之家″ + ″幸福院″ + ″老人院″ + ″敬老院″ + ″安老院″ + ″护理院″ + ″老年人公寓″) OR R=(″养老机构″ + ″养老院″ + ″护理之家″ + ″幸福院″ + ″老人院″ + ″敬老院″ + ″安老院″ + ″护理院″ + ″老年人公寓″)
#4	M=(″质性研究″ + ″定性研究″ + ″描述性研究″ + ″民族志″ + ″现象学″ + ″扎根理论″ + ″内容分析″ + ″主题分析″ + ″焦点小组″ + ″解释学″ + ″访谈″ + ″叙述″ + ″混合性研究″) OR R=(″质性研究″ + ″定性研究″ + ″描述性研究″ + ″民族志″ + ″现象学″ + ″扎根理论″ + ″内容分析″ + ″主题分析″ + ″焦点小组″ + ″解释学″ + ″访谈″ + ″叙述″ + ″混合性研究″)
#5	#1 AND #2 AND #3 AND #4

For peer review only

JBI Critical Appraisal Checklist for Qualitative Research

Reviewer____________________________________Date___________________________

Author ____________________________________Year_________Record Number______

	Yes	No	Unclear	Not applicable
1. Is there congruity between the stated philosophical perspective and the research methodology?	☐	☐	☐	☐
2. Is there congruity between the research methodology and the research question or objectives?	☐	☐	☐	☐
3. Is there congruity between the research methodology and the methods used to collect data?	☐	☐	☐	☐
4. Is there congruity between the research methodology and the representation and analysis of data?	☐	☐	☐	☐
5. Is there congruity between the research methodology and the interpretation of results?	☐	☐	☐	☐
6. Is there a statement locating the researcher culturally or theoretically?	☐	☐	☐	☐
7. Is the influence of the researcher on the research, and vice- versa, addressed?	☐	☐	☐	☐
8. Are participants, and their voices, adequately represented?	☐	☐	☐	☐
9. Is the research ethical according to current criteria or, for recent studies, and is there evidence of ethical approval by an appropriate body?	☐	☐	☐	☐
10. Do the conclusions drawn in the research report flow from the analysis, or interpretation, of the data?	☐	☐	☐	☐

Overall appraisal: Include ☐ Exclude ☐ Seek further info ☐

Comments (Including reason for exclusion)

____

____

____

JBI QARI Data Extraction Tool for Qualitative Research

Reviewer ______________________ Date ______________________

Author ______________________ Year ______________________

Journal ______________________ Record Number ______________________

Study Description

Methodology

____

Method

____

Phenomena of interest

____

Setting

____

Geographical

____

Cultural

____

Participants

____

Data analysis

____

Authors conclusions

____

Comments

____

Complete Yes ☐ No ☐

Copyright © The Jo0anna Briggs Institute 2014

Findings	Illustration form Publication (page number)	Evidence		
		Unequivocal	Credible	Unsupported

Extraction of findings complete Yes ☐ No ☐

Copyright © The Jo0anna Briggs Institute 2014

y only

Reporting checklist for protocol of a systematic review and meta analysis.

Based on the PRISMA-P guidelines.

Instructions to authors

Complete this checklist by entering the page numbers from your manuscript where readers will find each of the items listed below.

Your article may not currently address all the items on the checklist. Please modify your text to include the missing information. If you are certain that an item does not apply, please write "n/a" and provide a short explanation.

Upload your completed checklist as an extra file when you submit to a journal.

In your methods section, say that you used the PRISMA-Preporting guidelines, and cite them as:

Moher D, Shamseer L, Clarke M, Ghersi D, Liberati A, Petticrew M, Shekelle P, Stewart LA. Preferred Reporting Items for Systematic Review and Meta-Analysis Protocols (PRISMA-P) 2015 statement. Syst Rev. 2015;4(1):1.

		Reporting Item	Page Number
**Title**			
Identification	#1a	Identify the report as a protocol of a systematic review	P1 (Line2)
Update	#1b	If the protocol is for an update of a previous systematic review, identify as such	n/a

For peer review only

**Registration**			
	#2	If registered, provide the name of the registry (such as PROSPERO) and registration number	P2(Lines55-57)
**Authors**			
Contact	#3a	Provide name, institutional affiliation, e-mail address of all protocol authors; provide physical mailing address of corresponding author	P1(Lines4-19)
Contribution	#3b	Describe contributions of protocol authors and identify the guarantor of the review	P9-P10(Lines262-268)
**Amendments**			
	#4	If the protocol represents an amendment of a previously completed or published protocol, identify as such and list changes; otherwise, state plan for documenting important protocol amendments	n/a
**Support**			
Sources	#5a	Indicate sources of financial or other support for the review	P10(Lines269-271)
Sponsor	#5b	Provide name for the review funder and / or sponsor	n/a
Role of sponsor or funder	#5c	Describe roles of funder(s), sponsor(s), and / or institution(s), if any, in developing the protocol	n/a
**Introduction**			

For peer review only

Rationale	#6	Describe the rationale for the review in the context of what is already known	P3-P5(Lines73-121)
Objectives	#7	Provide an explicit statement of the question(s) the review will address with reference to participants, interventions, comparators, and outcomes (PICO)	P5(Lines122-125)
**Methods**			
Eligibility criteria	#8	Specify the study characteristics (such as PICO, study design, setting, time frame) and report characteristics (such as years considered, language, publication status) to be used as criteria for eligibility for the review	P5-P6(Lines135-151)
Information sources	#9	Describe all intended information sources (such as electronic databases, contact with study authors, trial registers or other grey literature sources) with planned dates of coverage	P6(Lines152-158)
Search strategy	#10	Present draft of search strategy to be used for at least one electronic database, including planned limits, such that it could be repeated	Supplementary File 1
Study records - data management	#11a	Describe the mechanism(s) that will be used to manage records and data throughout the review	P6-P7(Lines173-177)
Study records - selection process	#11b	State the process that will be used for selecting studies (such as two independent reviewers) through each phase of the review (that is, screening, eligibility and	P7-P9

For peer review only

		inclusion in meta-analysis)	
Study records - data collection process	#11c	Describe planned method of extracting data from reports (such as piloting forms, done independently, in duplicate), any processes for obtaining and confirming data from investigators	P7-P8(Lines199-214)
Data items	#12	List and define all variables for which data will be sought (such as PICO items, funding sources), any pre-planned data assumptions and simplifications	P7-P8(Lines202-207)
Outcomes and prioritization	#13	List and define all outcomes for which data will be sought, including prioritization of main and additional outcomes, with rationale	P9(Lines253-258)
Risk of bias in individual studies	#14	Describe anticipated methods for assessing risk of bias of individual studies, including whether this will be done at the outcome or study level, or both; state how this information will be used in data synthesis	P7(Lines188-198)
Data synthesis	#15a	Describe criteria under which study data will be quantitatively synthesised	n/a
Data synthesis	#15b	If data are appropriate for quantitative synthesis, describe planned summary measures, methods of handling data and methods of combining data from studies, including any planned exploration of consistency (such as I2, Kendall's τ)	n/a
Data synthesis	#15c	Describe any proposed additional analyses (such as	n/a

For peer review only

		sensitivity or subgroup analyses, meta-regression)	
Data synthesis	#15d	If quantitative synthesis is not appropriate, describe the type of summary planned	P8(Lines216-133)
Meta-bias(es)	#16	Specify any planned assessment of meta-bias(es) (such as publication bias across studies, selective reporting within studies)	P3(Lines70-71)
Confidence in cumulative evidence	#17	Describe how the strength of the body of evidence will be assessed (such as GRADE)	P8-P9(Lines234-242)

None The PRISMA-P elaboration and explanation paper is distributed under the terms of the Creative Commons Attribution License CC-BY. This checklist can be completed online using https://www.goodreports.org/, a tool made by the EQUATOR Network in collaboration with Penelope.ai

For peer review only

Barriers and facilitators to dementia care in long-term care facilities: Protocol for a qualitative systematic review and meta-synthesis

Journal:	*BMJ Open*
Manuscript ID	bmjopen-2023-076058.R1
Article Type:	Protocol
Date Submitted by the Author:	26-Sep-2023
Complete List of Authors:	Zhang, Xi; Xi'an Jiaotong University<br>Guan, Chengguo; Xi'an Jiaotong University<br>He, Jinjie; Xi'an Jiaotong University<br>Wang, Jing; Xi'an Jiaotong University
**Primary Subject Heading**:	Nursing
Secondary Subject Heading:	Geriatric medicine
Keywords:	Systematic Review, Dementia < NEUROLOGY, QUALITATIVE RESEARCH, Protocols & guidelines < HEALTH SERVICES ADMINISTRATION & MANAGEMENT

Barriers and facilitators to dementia care in long-term care facilities：Protocol for a qualitative systematic review and meta-synthesis

Authors and affiliation

Xi Zhang[1], Chengguo Guan[1], Jinjie He[1], Jing Wang[1]*

[1]Health Science Center, Xi'an Jiaotong University, Xi'an, Shaanxi, China

Email

Xi Zhang: zhangxi1234560814@163.com

Chengguo Guan: novogcg@stu.xjtu.edu.cn

Jinjie He: hjj521209@stu.xjtu.edu.cn

Jing Wang*: novowj@xjtu.edu.cn

***Corresponding author**

Jing Wang

Health Science Center, Xi'an Jiaotong University

No.76 Yanta West Road, Xi'an, Shaanxi, China

Phone: +86-18789400330

Email: novowj@xjtu.edu.cn

Keywords:

dementia, long-term care facilities, qualitative study, systematic review, protocol

Word count

2369

For peer review only

ABSTRACT

Introduction: Long-term care needs for people with dementia are predicted to increase due to increased life expectancy and dementia diagnoses. Most published meta-synthesis of dementia care focus on hospital or home settings, and about long-term care facilities, most reviews about dementia care only focus on a single outcome, such as feeding, behavioral symptoms management, palliative care, and others, which is limited. The present study aims to synthesise qualitative data and examine barriers and facilitators to caring for people with dementia in long-term care facilities.

Methods and analysis: This is the protocol for our systematic review and meta-synthesis, which describes the design of this study, and we plan to complete the study from October 2023 to November 2024. The systematic review and meta-synthesis will follow the Joanna Briggs Institute (JBI) guidance for systematic reviews of qualitative evidence. Nine databases (five English and four Chinese) were searched, including Embase, Web of Science, Medline, CINAHL, PsycINFO and Wan Fang Data, China National Knowledge Infrastructure (CNKI), VIP, and Chinese Biomedical Medicine (CBM), from inception to August 2023. Qualitative and mixed-approach researches about barriers and facilitators to caring for people with dementia in long-term care facilities, which are reported in English or Chinese, will be included. Covidence software will help with study selection, assessment, and data extraction. The JBI Critical Appraisal Checklist for Qualitative Research (2020) will be used for included studies' quality assessment. Data extraction will be based on the JBI-QARI Data Extraction Tool for Qualitative Research. The JBI aggregation approach will be used to synthesises data. We will use the JBI ConQual tool to assess the credibility and dependability of each synthesised finding to establish confidence in the synthesized findings. All review steps will be managed by two reviewers independently, disparities will be discussed. If consensus cannot reach a resolution, a third reviewer will be consulted.

Ethics and dissemination: The present study is a secondary analysis of published qualitative data. So ethical approval is not required. The findings may be disseminated

For peer review only

through peer-reviewed publications, conference papers or elsewhere.

Registration details: The protocol was registered with the International Prospective Register of Systematic Reviews (PROSPERO) in May 2022, and the registration number is CRD42022326178.

Keywords: dementia, long-term care facilities, qualitative study, systematic review, protocol

ARTICLE SUMMARY

Strengths and limitations of this study

- This study is the first systematic review and meta-synthesis to synthesize qualitative evidence that explores the factors of caring for people with dementia in long-term care facilities.
- This study will be guided by the Joanna Briggs Institute methodology for qualitative systematic reviews to increase the accuracy and reliability of results.
- A limitation is the exclusion of studies not written in English or Chinese, and of grey literature.

INTRODUCTION

Dementia is a degenerative syndrome affecting individuals' cognitive functions. It impacts memory, behavior, thinking, and social abilities as the disease advances. Those affected become increasingly dependent on care from others for all activities of daily living.[1] Previous studies show that age is a critical factor in dementia, and there is no cure for dementia currently.[2] The demographic shift to an aging population has rapidly increased the number of people with dementia. According to the most recent World Health Organization (WHO) figures, 55.2 million people worldwide were living with dementia in 2019, and the number is expected to rise to 139 million in 2050.[3] Caring for people with dementia requires round-the-clock care, and caregivers must manage behavioral and psychological symptoms (such as aggression, wandering, and depression), placing an overwhelming burden on the caregivers' well-being, especially

For peer review only

in the severe stage of dementia.[4, 5] Long-term care facilities are the alternative place when people with dementia cannot get sufficient support at home.[6, 7] Long-term care facilities may have different names in different countries, like nursing homes, skilled nursing facilities, assisted living facilities, and residential facilities, providing services to people who cannot live independently.[8] Approximately over 30% of long-term care facility residents have a form of dementia in higher-income economies.[3] In lower- and middle-income countries, informal caregivers, mostly family members, provide a significant proportion of dementia care nowadays.[3] However, long-term care needs for people with dementia are predicted to increase due to increased life expectancy and dementia diagnoses.

The WHO Framework for Countries to achieve an integrated continuum of long-term care recognizes critical aspects for an integrated continuum service provision in long-term care facilities and guides countries in evaluating and implementing equitable and sustainable care in long-term care facilities.[9] The goal of long-term care is to ensure that an individual who has significant declines in physical or mental capacity can maintain the best possible quality of life, with the greatest possible degree of independence, autonomy, participation, personal fulfillment and human dignity.[9] Professionals need to take a holistic approach to providing care to the people living with dementia, depending on the situation, dementia care should include basic care such as eating and dressing, pain management, behavioral and psychological symptoms management, and palliative care to promote their well-being and quality of life.[10] But now, knowledge-practice gaps about dementia care in long-term care facilities have been still revealed by several studies: the quality of life still need to be improved;[11] feeding difficulties,[12] pain management,[13] and other disease-related care needs require further attention. Besides, the behavioral and psychological symptoms are common in people with dementia, and how to manage these symptoms is still an endeavor.[14] Professionals, as key service providers in long-term care facilities, face many difficulties in providing care for people with dementia.[15-18] Some qualitative studies suggest that professionals find it hard to provide personalized care to patients and interact with them in long-term care facilities because of insufficient time and a

For peer review only

lack of knowledge about dementia.[15, 17] It has also been noted that when a professional's attitude towards dementia is positive and hopeful, the more it contributes to the caring of people with dementia.[19] In addition to exploring the experiences of professionals in holistic dementia care, there are also studies that focus on a single outcome. Some studies suggest that management of behavioral and psychological symptoms may be limited by lack of clinical guidance and resources,[20] and feeding care is also associated with clinical resources, in addition to the environment.[21]

In recent years, qualitative studies are proliferating in nursing research because they effectively complement quantitative research results.[22] Qualitative studies explore detailed explanations and meanings shown by participants, providing an alternative perspective to a more positivist approach.[23] Meta-synthesis of qualitative studies can yield broader results because they synthesize a more comprehensive range of participants and descriptions. It integrates the body of knowledge about a topic and, as a result of this process, can produce new interpretations of the phenomenon that contribute to understanding the topic.[24] To date, a certain number of qualitative studies have explored the experience of dementia care from the perspective of professionals. There has been some meta-analysis based on these articles, but most published meta-synthesis of dementia care focus on hospital[25-27] or home settings.[28-30] Regarding long-term care facilities, most reviews about dementia care only focus on a single outcome, such as feeding,[31, 32] behavioral symptoms management,[33, 34] palliative care,[35] and others, which is limited. The dementia care is holistic and considering only one aspect of care is not comprehensive. Integrating these known factors to identify barriers and facilitators to dementia care in long-term care facilities is essential.

To the best of our knowledge, there are currently no studies that have systematically evaluated qualitative research on holistic dementia care in long-term care facilities. Therefore, the present study aims to systematically review and synthesise the best available qualitative evidence on barriers and facilitators to caring for people with dementia in long-term care facilities from the perspective of the professionals, providing more comprehensive evidence for better service development in dementia

For peer review only

care in long-term care facilities.

METHOD AND ANALYSIS

Design

The protocol was registered with the International Prospective Register of Systematic Reviews (PROSPERO) in May 2022, and the registration number is CRD42022326178. This protocol was designed following the Preferred reporting items for systematic review and meta-analysis protocols (PRISMA-P) 2015 statement[36, 37] and the Joanna Briggs Institute (JBI) guidance for systematic reviews of qualitative evidence.[38] The study will run from October 2023 to November 2024.

Eligibility criteria

Type of participants: Studies focusing on health professionals (aged≥18) caring for people with dementia in long-term care facilities, including doctors, nurses, and other health professionals. Studies focusing on multiple disease types (not only care for people with dementia) will also be excluded.

Phenomena of interest: Studies focusing on barriers and facilitators to caring for people with dementia in long-term care facilities, including experiences in basic care such as eating and dressing, pain management, behavioral and psychological symptoms management, palliative care and other related care.

Context: Studies focusing on long-term care facilities,[8] such as nursing homes, skilled nursing facilities, assisted living facilities, nursing institutes, and residential facilities, providing services to people who cannot live independently. Studies conducted in adult daycare facilities, respite care, and other transitional care facilities will also be included.

Types of studies: Qualitative and mixed methods studies with an analytical part of the qualitative content published in English or Chinese will be included. Only the qualitative component of the mixed methods studies will be considered. Quantitative studies, pilot studies, reviews, case reports, case series, reanalysis of data, conference abstracts, and expert opinions will be excluded.

Information sources

For peer review only

Nine databases will be searched, including five English databases and four Chinese databases with no time limit applied: The five English databases are Embase, Web of Science, Medline, CINAHL, PsycINFO and the four Chinese databases are Wan Fang Data, China National Knowledge Infrastructure (CNKI), VIP and Chinese Biomedical Medicine (CBM). References and citations of the included studies will also be searched to identify if there are any other relevant sources.

Search strategy

Keywords were first formed based on the eligibility criteria, and synonyms of each keyword were then analysed to create a logic grid to capture relevant studies. Boolean operators will be used to combine all the keywords. Pilot searches will be conducted to confirm the sensitivity and specificity of the strategy in all databases before the formal search is conducted. The final strategy will be consulted by an expert librarian and reviewed by all team members. The index terms and the search strategy were developed in Web of Science among five English databases and China National Knowledge Infrastructure among four Chinese databases. The Web of Science English search strategy and China National Knowledge Infrastructure Chinese search strategy will be then translated and used for additional databases. We will include studies retrievable from the establishment of the database to August 2023. Search strategies are available in Supplement 1. Besides, citation tracing will be conducted, the reference lists of included studies will be screened to identify studies that were missed in the search process.

Study selection

All search records will be imported to Endnote X9 (V.9.3.3) for duplicates removal and then to Covidence software (https://app.covidence.org/reviews/active) for study selection. Covidence is a systematic review production tool developed by researchers specialized in the systematic review process to standardize the process of conducting systematic reviews. According to the proposed eligibility criteria, title and abstract screening will be conducted by two reviewers (CG and XZ) independently on all records. Then, the full-text review of included studies will be screened in the same way,

For peer review only

and the reason for exclusion will be selected and recorded in Covidence. Before each formal process, the pilot test will be conducted initially with 5-10 percent of studies screened by all reviewers independently to facilitate understanding and application of eligibility criteria and the tool. Disparities will be discussed during the screening process via group discussion. If a resolution cannot be reached by consensus, the third and fourth reviewers (JH and JW) will be consulted. Regular group meetings with all the reviewers will be held regularly to discuss and resolve any disagreements. The results of the screening will be recorded in a PRISMA flow chart.

Risk of bias assessment

The JBI Critical Appraisal Checklist for Qualitative Research (2020) (Supplement 2) will be used for assessing the quality of included studies, which includes ten items that assess the studies' philosophical foundation, methodology, method of data collection, method of data analysis, results interpretation, the logic of studies and research ethics. The four responses "yes," "no," "unclear," and "not applicable" will be used to evaluate each item. The final quality rating of the study would be rated as high, medium, or low based on the assessment of individual items and the amount of weight given to the degree to which each item impacted the findings of the study.[38, 39] Only studies having at least a medium ranking will be included. Two reviewers (CG and XZ) will appraise all included studies' methodological quality independently and disparities will be discussed. The third and fourth reviewers (JH and JW) will be consulted when a resolution cannot be reached by consensus.

Data extraction

Data extraction will be conducted based on the JBI Qualitative Assessment and Review Instrument (JBI-QARI) Data Extraction Tool for Qualitative Research (Supplement 3) by two reviewers (CG and XZ) independently.[38] According to the tool, the characteristics of included studies and findings with illustrations will be extracted. Information will be extracted using Covidence, including (1) author, publication, year, country, geographical location, and study setting; (2) participants' characteristics and sample size; (3) research design, methodology, and methods; (4) phenomena of interest and main findings. Before the commencement of the formal data extraction, data of 5-

For peer review only

10 percent included studies will be extracted using the data extraction tool to pilot test the tool and minimize the risk of error. According to the JBI review manual, all the findings extracted from the selected studies will be evaluated and divided into three levels based on the degree of support between the finding and its illustration: unequivocal, credible and unsupported.[38] Only the findings rated as unequivocal or credible will be included for data synthesis. Any disagreements between the two reviewers will be discussed and resolved by regular team meetings with the four reviewers.

Data synthesis

The JBI meta aggregation approach with a three-step process will be used for data synthesis after data extraction.[40] In the three-step data synthesis process, the findings will first be extracted from the original studies, then the included findings will be grouped into categories based on similarity of meaning, and similar categories will ultimately be further synthesized into different synthesized findings based on their relationships and relevance to the research objective of this review. This approach focuses on the essence of qualitative research and emphasizes the value and role of qualitative research in an evidence-based healthcare service system, collecting all included studies' findings and summarizing them according to their meanings to make them more targeted, convincing, and generalizing. On the premise of understanding the philosophical foundation and methodology of various qualitative studies, reviewers repeatedly read, understand, analyze, and interpret the meaning of each included findings, combine similar findings, form a new category, and finally summarize the category as synthesized findings. In the present review, the findings of each included study will be read carefully to understand completely before data synthesis. Regular meetings will be held with the four reviewers to reach an agreement on the final synthesized findings, and any conflicting opinions between the reviewers will be settled by thorough discussions.

Assessing the confidence of findings

We will use the JBI ConQual tool to assess the confidence of each synthesised finding.[41] Dependability and credibility are two elements to influence the confidence

For peer review only

of qualitative synthesised findings. Dependability can be established by examining the quality of the original studies included through a set of critical appraisal questions. Credibility evaluates the goodness of fit between the author's interpretation and the original data. First, the confidence of synthesised findings will be initially assumed to be graded as high, and then downgraded based on the results of dependability and credibility assessments, and finally each synthesised finding will be rated high, moderate, low, or very low.[41]

Patient and public involvement

None.

Ethics and dissemination

The present study is a secondary analysis of published qualitative data; there is no concern for privacy. So ethical approval is not required for the present study. However, the research ethics of each included original study will be assessed rigorously. The findings may be disseminated through peer-reviewed publications, conference papers or elsewhere.

Acknowledgments

We gratitude for the help and support of the National Social Science Fund of China.

Author Contributions

JW and CG conceived the topic of this study. All team members (JW, CG, JH, XZ) participated in the discussion of the study design and made significant comments. JW and CG constructed the search strategy. CG, JH, XZ, and JW will conduct data selection, assessment, extraction, and synthesis. XZ drafted the protocol manuscript, and then the manuscript was reviewed by JW. All authors approved the submitted protocol and are accountable for its content.

Funding statement

This work was supported by The National Social Science Fund of China (Grant No. 20XRK004).

Competing interests

none

For peer review only

References

1. World Alzheimer Report 2021: Journey through the diagnosis of dementia. *Alzheimer's Disease International,* 2021. https://www.alzint.org/resource/world-alzheimer-report-2021/.

2. Jia L, Du Y, Chu L, et al. Prevalence, risk factors, and management of dementia and mild cognitive impairment in adults aged 60 years or older in China: a cross-sectional study. *Lancet Public Health* 2020;5(12):e661-e71. doi: 10.1016/s2468-2667(20)3015-7

3. WHO Global status report on the public health response to dementia Executive summary. *World Health Organization (WHO),* 2021. https://www.who.int/multi-media/details/global-status-report-on-the-public-health-response-to-dementia.

4. Wang J, Xiao LD, Li X, et al. Caregiver distress and associated factors in dementia care in the community setting in China. *Geriatr Nurs* 2015;36(5):348-54. doi: 10.1016/j.gerinurse.2015.04.013

5. Connors MH, Seeher K, Teixeira-Pinto A, et al. Dementia and caregiver burden: A three-year longitudinal study. *Int J Geriatr Psychiatry* 2020;35(2):250-58. doi: 10.1002/gps.5244

6. Xiao LD, Wang J, He GP, et al. Family caregiver challenges in dementia care in Australia and China: a critical perspective. *BMC Geriatr* 2014;14:6. doi: 10.1186/1471-2318-14-6

7. Gaugler JE, Mittelman MS, Hepburn K, et al. Predictors of change in caregiver burden and depressive symptoms following nursing home admission. *Psychol Aging* 2009;24(2):385-96. doi: 10.1037/a0016052

8. Preventing and managing COVID-19 across long-term care services: Policy brief, 24 July 2020. *world Health Organization (WHO),* 2020. https://www.who.int/publications/i/item/WHO-2019-nCoV-Policy_Brief-Long-term_Care-2020.1.

9. Framework for countries to achieve an integrated continuum of long-term care. *World Health Organization (WHO)* , 2021.

For peer review only

https://www.who.int/publications/i/item/9789240038844.

10. Fazio S, Pace D, Maslow K, et al. Alzheimer's Association Dementia Care Practice Recommendations. *Gerontologist* 2018;58(suppl_1):S1-s9. doi: 10.1093/geront/gnx182

11. Olsen C, Pedersen I, Bergland A, et al. Differences in quality of life in home-dwelling persons and nursing home residents with dementia - a cross-sectional study. *BMC Geriatr* 2016;16:137. doi: 10.1186/s12877-016-0312-4

12. Perry E, Walton K, Lambert K. Prevalence of Malnutrition in People with Dementia in Long-Term Care: A Systematic Review and Meta-Analysis. *Nutrients* 2023;15(13). doi: 10.3390/nu15132927

13. Cole CS, Carpenter JS, Chen CX, et al. Prevalence and Factors Associated with Pain in Nursing Home Residents: A Systematic Review of the Literature. *J Am Med Dir Assoc* 2022;23(12):1916-25.e1. doi: 10.1016/j.jamda.2022.08.008

14. O'Donnell E, Holland C, Swarbrick C. Strategies used by care home staff to manage behaviour that challenges in dementia: A systematic review of qualitative studies. *Int J Nurs Stud* 2022;133:104260. doi: 10.1016/j.ijnurstu.2022.104260

15. Barbosa A, Nolan M, Sousa L, et al. Dementia in Long-term Care Homes: Direct Care Workers' Difficulties. *Procedia - Social and Behavioral Sciences* 2014;140:172-77. org/10.1016/j.sbspro.2014.04.405

16. Zhao Y, Liu L, Ding Y, et al. Understanding dementia care in care home setting in China: An exploratory qualitative study. *Health Soc Care Community* 2021;29(5):1511-21. doi: 10.1111/hsc.13213

17. Kong EH, Kim H, Kim H. Nursing home staff's perceptions of barriers and needs in implementing person-centred care for people living with dementia: A qualitative study. *J Clin Nurs* 2022;31(13-14):1896-906. doi: 10.1111/jocn.15729

18. Law K, Patterson TG, Muers J. Experiences of healthcare assistants working with clients with dementia in residential care homes. *Dementia (London)* 2019;18(2):644-59. doi: 10.1177/1471301216688396

19. Gerritsen DL, van Beek APA, Woods RT. Relationship of care staff attitudes with social well-being and challenging behavior of nursing home residents with dementia: a

For peer review only

cross sectional study. *Aging Ment Health* 2019;23(11):1517-23. doi: 10.1080/13607863.2018.1506737

20. Jennings AA, Foley T, McHugh S, et al. 'Working away in that Grey Area…' A qualitative exploration of the challenges general practitioners experience when managing behavioural and psychological symptoms of dementia. *Age Ageing* 2018;47(2):295-303. doi: 10.1093/ageing/afx175

21. Sadarangani T, Chong S, Park S, et al. A Qualitative Analysis of the Delivery of Person-Centered Nutrition to Asian Americans With Dementia in the Adult Day Health Care Setting. *J Appl Gerontol* 2021;40(2):179-88. doi: 10.1177/0733464820910030

22. Wang J, Li X, Liu W, et al. The positive aspects of caregiving in dementia: A scoping review and bibliometric analysis. *Front Public Health* 2022;10:985391. doi: 10.3389/fpubh.2022.985391

23. Lindgren BM, Lundman B, Graneheim UH. Abstraction and interpretation during the qualitative content analysis process. *Int J Nurs Stud* 2020;108:103632. doi: 10.1016/j.ijnurstu.2020.103632

24. Evans D, Pearson A. Systematic reviews of qualitative research. *Clinical Effectiveness in Nursing* 2005;5:111-19. doi: 10.1054/cein.2001.0219

25. Brossard Saxell T, Ingvert M, Lethin C. Facilitators for person-centred care of inpatients with dementia: A meta-synthesis of registered nurses' experiences. *Dementia (London)* 2021;20(1):188-212. doi: 10.1177/1471301219871408

26. Moonga J, Likupe G. A systematic literature review on nurses' and health care support workers' experiences of caring for people with dementia on orthopaedic wards. *J Clin Nurs* 2016;25(13-14):1789-804. doi: 10.1111/jocn.13158

27. Digby R, Lee S, Williams A. The experience of people with dementia and nurses in hospital: an integrative review. *J Clin Nurs* 2017;26(9-10):1152-71. doi: 10.1111/jocn.13429

28. Köhler K, Dreyer J, Hochgraeber I, et al. Towards a middle-range theory of 'Stability of home-based care arrangements for people living with dementia' (SoCA-Dem): findings from a meta-study on mixed research. *BMJ Open* 2021;11(4):e042515. doi: 10.1136/bmjopen-2020-042515

For peer review only

29. Dawson A, Bowes A, Kelly F, et al. Evidence of what works to support and sustain care at home for people with dementia: a literature review with a systematic approach. *BMC Geriatr* 2015;15:59. doi: 10.1186/s12877-015-0053-9

30. Marulappa N, Anderson NN, Bethell J, et al. How to implement person-centred care and support for dementia in outpatient and home/community settings: Scoping review. *BMC Health Serv Res* 2022;22(1):541. doi: 10.1186/s12913-022-07875-w

31. Li Y, Zhang X, Su J, et al. Optimizing mealtime care for people with dementia from the perspective of formal caregivers: A systematic review of qualitative research. *Int J Nurs Stud* 2021;123:104046. doi: 10.1016/j.ijnurstu.2021.104046.

32. Faraday J, Salis C, Barrett A. Equipping Nurses and Care Staff to Manage Mealtime Difficulties in People With Dementia: A Systematic Scoping Review of Training Needs and Interventions. *Am J Speech Lang Pathol* 2019;28(2):717-42. doi: 10.1044/2018_ajslp-18-0062

33. Walsh KA, Dennehy R, Sinnott C, et al. Influences on Decision-Making Regarding Antipsychotic Prescribing in Nursing Home Residents With Dementia: A Systematic Review and Synthesis of Qualitative Evidence. *J Am Med Dir Assoc* 2017;18(10):897.e1-97.e12. doi: 10.1016/j.jamda.2017.06.032

34. Jennings AA, Foley T, Walsh KA, et al. General practitioners' knowledge, attitudes, and experiences of managing behavioural and psychological symptoms of dementia: A mixed-methods systematic review. *Int J Geriatr Psychiatry* 2018;33(9):1163-76. doi: 10.1002/gps.4918

35. van der Steen JT, Radbruch L, Hertogh CM, et al. White paper defining optimal palliative care in older people with dementia: a Delphi study and recommendations from the European Association for Palliative Care. *Palliat Med* 2014;28(3):197-209. doi: 10.1177/0269216313493685

36. Moher D, Shamseer L, Clarke M, et al. Preferred reporting items for systematic review and meta-analysis protocols (PRISMA-P) 2015 statement. *Syst Rev* 2015;4(1):1. doi: 10.1186/2046-4053-4-1

37. Shamseer L, Moher D, Clarke M, et al. Preferred reporting items for systematic review and meta-analysis protocols (PRISMA-P) 2015: elaboration and explanation.

Bmj 2015;350:g7647. doi: 10.1136/bmj.g7647

38. Lockwood C, Porritt K, Munn Z, et al. Chapter 2: Systematic reviews of qualitative evidence. *JBI Manual for Evidence Synthesis*; JBI, 2020. https://synthesismanual.jbi.global.

39. Ying G, Huiwen Z, Yingfeng Z, et cl. JBI evidence-based health center's quality assessment tool for different types of research- The quality evaluation of systematic review. *Journal of Nurses Training*, 2018;33(08):701-703. doi:10.16821/j.cnki.hsjx.2018.08.008.

40. Lockwood C, Munn Z, Porritt K. Qualitative research synthesis: methodological guidance for systematic reviewers utilizing meta-aggregation. *Int J Evid Based Healthc* 2015;13(3):179-87. doi: 10.1097/xeb.0000000000000062

41. Munn Z, Porritt K, Lockwood C, et al. Establishing confidence in the output of qualitative research synthesis: the ConQual approach. *BMC Med Res Methodol* 2014;14:108. doi: 10.1186/1471-2288-14-108

For peer review only

Barriers and facilitators to dementia care in long-term care facilities: A systematic review and meta-synthesis protocol

Supplement file 1 - Searched databases and search strategies

1. Search strategy for Web of Science	
#1	**TI=**(dement* OR "presenile dementia" OR "cognitive impairment" OR "cognitive dysfunction" OR "Alzheimer's disease" OR "Alzheimer's dementia" OR AD OR "mild cognitive impairment" OR "huntington disease" OR "lewy body" OR "chronic cerebrovascular" OR "parkinson disease dementia" OR "parkinson dementia" OR “lewy bodies” OR “lewy neurites” OR “dementia, vascular” OR “vascular dementia*”) OR **AB=**(dementia OR "presenile dementia" OR "cognitive impairment" OR "cognitive dysfunction" OR "Alzheimer's disease" OR "Alzheimer's dementia" OR AD OR "mild cognitive impairment" OR "huntington disease" OR "lewy body" OR "chronic cerebrovascular" OR "parkinson disease dementia" OR "parkinson dementia" OR “lewy bodies” OR “lewy neurites” OR “dementia, vascular” OR “vascular dementia*”)
#2	**TI=**(nursing OR "practical nursing" OR "care, nursing" OR "management, nursing Care" OR "care, long-term" OR "long term care" OR care OR caring OR caregiving OR manag* OR "long-term care") OR **AB=**(nursing OR "practical nursing" OR "care, nursing" OR "management, nursing Care" OR "care, long-term" OR "long term care" OR care OR caring OR caregiving OR manag* OR "long-term care")
#3	**TI=**("elderly care institution" OR "nursing home*" OR "health care setting" OR "long term care facilities" OR "residential aged care" OR "care home*" OR "residential facilities" OR "homes for aged" OR "residential home*" OR "residential facilit*" OR "nursing home facility*" OR "nursing home care" OR "nursing home placement" OR "old people's home*" OR "homes for the aged" OR "old age home" OR "old age homes" OR "housing for the elderly") OR **AB=**("elderly care institution" OR "nursing home*" OR "health care setting" OR "long term care facilities" OR "residential aged care" OR "care home*" OR "residential facilities" OR "homes for aged" OR "residential home*" OR "residential facilit*" OR "nursing home facility*" OR "nursing home care" OR "nursing home placement" OR "old people's home*" OR "homes for the aged" OR "old age home" OR "old age homes" OR "housing for the elderly")
#4	**TI=**("nursing methodology" OR "case study" OR "constant comparison" OR "content analysis" OR "descriptive study" OR "discourse analysis" OR ethnography OR exploratory OR feminist OR "focus group*" OR "grounded theory" OR hermeneutic OR interview OR narrative OR naturalistic OR "participant observation" OR phenomenology OR "qualitative method" OR "qualitative research" OR "qualitative study" OR "thematic analysis" OR "mixed method research" OR "mixed methods research") OR **AB=**("nursing methodology" OR "case study" OR "constant comparison" OR "content analysis" OR "descriptive study" OR "discourse analysis" OR ethnography OR exploratory OR feminist OR "focus group*" OR "grounded theory" OR hermeneutic OR interview OR narrative OR naturalistic OR "participant observation" OR phenomenology OR "qualitative method" OR "qualitative research" OR

For peer review only

	"qualitative study" OR "thematic analysis" OR "mixed method research" OR "mixed methods research")
#5	#1 AND #2 AND #3 AND #4
2. Search strategy for Embase	
#1	**'Alzheimer disease'/exp OR 'dementia'/exp OR 'mild cognitive impairment'/exp OR 'Lewy body'/exp OR 'multiinfarct dementia'/exp**
#2	dement*:**ti,ab** OR 'presenile dementia':ti,ab OR 'cognitive impairment':ti,ab OR alzheimer*:ti,ab OR ad:ti,ab OR 'mild cognitive impairment':ti,ab OR 'huntington disease':ti,ab OR 'lewy body':ab OR 'chronic cerebrovascular':ti,ab OR 'parkinson disease dementia':ti,ab OR 'parkinson dementia':ti,ab OR 'cognitive dysfunction':ti,ab OR 'lewy bodies':ti,ab OR 'lewy neurites':ti,ab OR 'dementia, vascular':ti,ab OR 'multiinfarct dementia': ti,ab
#3	#1 OR #2
#4	**'management'/exp OR 'nursing'/exp OR 'practical nursing'/exp OR 'care'/exp OR 'custodial care'/exp OR 'nursing care'/exp OR 'long term care'/exp**
#5	nursings:**ti,ab** OR 'practical nursing':ti,ab OR 'nursing care':ti,ab OR 'management, nursing care':ti,ab OR 'care, long-term':ti,ab OR 'long term care':ti,ab OR care:ti,ab OR caring:ti,ab OR caregiving:ti,ab OR manag*:ti,ab OR 'long-term care':ti,ab
#6	#4 OR #5
#7	**'nursing home'/exp OR 'residential home'/exp OR 'home for the aged'/exp**
#8	'elderly care institution':**ti,ab** OR 'health care setting':ti,ab OR 'long term care facilities':ti,ab OR 'residential aged care':ti,ab OR 'care home*':ti,ab OR 'nursing home*':ti,ab OR 'residential facilities':ti,ab OR 'homes for aged':ti,ab OR 'residential home*':ti,ab OR 'residential facilit*':ti,ab OR 'nursing home facility*':ti,ab OR 'nursing home care':ti,ab OR 'nursing home placement':ti,ab OR 'homes for the aged':ti,ab OR 'old age home':ti,ab OR 'old age homes':ti,ab OR 'housing for the elderly':ti,ab
#9	#7 OR #8
#10	**'qualitative research'/exp**
#11	'nursing methodology':**ti,ab** OR 'case study':ti,ab OR 'constant comparison':ti,ab OR 'content analysis':ti,ab OR 'descriptive study':ti,ab OR 'discourse analysis':ti,ab OR ethnography:ti,ab OR exploratory:ti,ab OR feminist:ti,ab OR 'focus group*':ti,ab OR 'grounded theory':ti,ab OR hermeneutic:ti,ab OR interview:ti,ab OR narrative:ti,ab OR naturalistic:ti,ab OR 'participant observation':ti,ab OR phenomenology:ti,ab OR 'qualitative method':ti,ab OR 'qualitative research':ti,ab OR 'qualitative study':ti,ab OR 'thematic analysis':ti,ab OR 'mixed method research':ti,ab OR 'mixed methods research':ti,ab
#12	#10 OR #11
#13	#3 AND #6 AND #9 AND #12 AND
3. Search strategy for Medline	
#1	**exp Dementia/ or exp Frontotemporal Dementia/ or exp Dementia, Vascular/ or Cognitive Dysfunction/ or exp Lewy Bodies/ or exp Parkinson Disease/**
#2	(dement* or "presenile dementia" or "cognitive impairment" or "cognitive dysfunction" or "Alzheimer's disease" or "Alzheimer's dementia" or AD or "mild cognitive impairment" or "huntington disease" or "lewy body" or "chronic cerebrovascular" or "parkinson disease dementia" or "parkinson dementia" or "parkinson dementia").tw.

For peer review only

#3	#1 or #2
#4	**exp Nursing/ or exp Nursing, Practical/ or exp Custodial Care/ or Nursing Care/ or exp Long-Term Care/**
#5	(nursing or "practical nursing" or "care, nursing" or "management, nursing Care" or "care, long-term" or "long term care" or care or caring or caregiving or manag* or "long-term care").tw.
#6	#4 or #5
#7	**exp Nursing Homes/ or exp Residential Facilities/ or Homes for the Aged/**
#8	("elderly care institution" or "nursing home*" or "health care setting" or "long term care facilities" or "residential aged care" or "care home*" or "residential facilities" or "homes for aged" or "residential home*" or "residential facilit*" or "nursing home facility*" or "nursing home care" or "nursing home placement" or "old people's home*" or "homes for the aged" or "old age home" or "old age homes" or "housing for the elderly").tw.
#9	#7 or #8
#10	**exp Qualitative Research/**
#11	("nursing methodology" or "case study" or "constant comparison" or "content analysis" or "descriptive study" or "discourse analysis" or ethnography or exploratory or feminist or "focus group*" or "grounded theory" or hermeneutic or interview or narrative or naturalistic or "participant observation" or phenomenology or "qualitative method" or "qualitative research" or "qualitative study" or "thematic analysis" or "mixed method research" or "mixed methods research").tw.
#12	#10 or #11
#13	#3 and #6 and #9 and #12
4. Search strategy for CINAHL	
#1	**(MH "Dementia+") OR (MH "Frontotemporal Dementia+") OR (MH "Dementia, Vascular+") OR (MH "Lewy Body Disease") OR (MH "Mild Cognitive Impairment")**
#2	**TI** (dement* OR "presenile dementia" OR "cognitive impairment" OR "cognitive dysfunction" OR "Alzheimer's disease" OR "Alzheimer's dementia" OR AD OR "mild cognitive impairment" OR "huntington disease" OR "lewy body" OR "chronic cerebrovascular" OR "parkinson disease dementia" OR "parkinson dementia" OR “lewy bodies” OR “lewy neurites” OR “dementia, vascular” OR “vascular dementia*”)
#3	**AB** (dement* OR "presenile dementia" OR "cognitive impairment" OR "cognitive dysfunction" OR "Alzheimer's disease" OR "Alzheimer's dementia" OR AD OR "mild cognitive impairment" OR "huntington disease" OR "lewy body" OR "chronic cerebrovascular" OR "parkinson disease dementia" OR "parkinson dementia" OR “lewy bodies” OR “lewy neurites” OR “dementia, vascular” OR “vascular dementia*”)
#4	#1 OR #2 OR #3
#5	**(MH "Caring+") OR (MH "Long Term Care") OR (MH "Management+")**
#6	**TI** (nursing OR "practical nursing" OR "care, nursing" OR "management, nursing Care" OR "care, long-term" OR "long term care" OR care OR caring OR caregiving OR manag* OR "long-term care")
#7	**AB** (nursing OR "practical nursing" OR "care, nursing" OR "management, nursing Care" OR "care, long-term" OR "long term care" OR care OR caring OR caregiving OR manag* OR "long-term care")

#8	#5 OR #6 OR #7
#9	**(MH "Nursing Homes+") OR (MH "Residential Facilities+")**
#10	**TI** ("elderly care institution" OR "nursing home*" OR "health care setting" OR "long term care facilities" OR "residential aged care" OR "care home*" OR "residential facilities" OR "homes for aged" OR "residential home*" OR "residential facilit*" OR "nursing home facility*" OR "nursing home care" OR "nursing home placement" OR "old people's home*" OR "homes for the aged" OR "old age home" OR "old age homes" OR "housing for the elderly")
#11	**AB** ("elderly care institution" OR "nursing home*" OR "health care setting" OR "long term care facilities" OR "residential aged care" OR "care home*" OR "residential facilities" OR "homes for aged" OR "residential home*" OR "residential facilit*" OR "nursing home facility*" OR "nursing home care" OR "nursing home placement" OR "old people's home*" OR "homes for the aged" OR "old age home" OR "old age homes" OR "housing for the elderly")
#12	#9 OR #10 OR #11
#13	**(MH "Qualitative Studies+")**
#14	**TI** ("nursing methodology" OR "case study" OR "constant comparison" OR "content analysis" OR "descriptive study" OR "discourse analysis" OR ethnography OR exploratory OR feminist OR "focus group*" OR "grounded theory" OR hermeneutic OR interview OR narrative OR naturalistic OR "participant observation" OR phenomenology OR "qualitative method" OR "qualitative research" OR "qualitative study" OR "thematic analysis" OR "mixed method research" OR "mixed methods research")
#15	**AB** ("nursing methodology" OR "case study" OR "constant comparison" OR "content analysis" OR "descriptive study" OR "discourse analysis" OR ethnography OR exploratory OR feminist OR "focus group*" OR "grounded theory" OR hermeneutic OR interview OR narrative OR naturalistic OR "participant observation" OR phenomenology OR "qualitative method" OR "qualitative research" OR "qualitative study" OR "thematic analysis" OR "mixed method research" OR "mixed methods research")
#16	#13 OR #14 OR #15
#17	#4 AND #8 AND #12 AND #16
5. Search strategy for PsycINFO	
#1	**(DE "Dementia") OR (DE "Dementia with Lewy Bodies") OR (DE "Vascular Dementia") OR (DE "Alzheimer's Disease") OR (DE "Cognitive Impairment") OR (DE "Mild Cognitive Impairment")**
#2	**TI** (dement* OR "presenile dementia" OR "cognitive impairment" OR "cognitive dysfunction" OR "Alzheimer's disease" OR "Alzheimer's dementia" OR "AD" OR "mild cognitive impairment" OR "huntington disease" OR "lewy body" OR "chronic cerebrovascular" OR "parkinson disease dementia" OR "parkinson dementia" OR “lewy bodies” OR “lewy neurites” OR “dementia, vascular” OR “vascular dementia*”)
#3	**AB** (dement* OR "presenile dementia" OR "cognitive impairment" OR "cognitive dysfunction" OR "Alzheimer's disease" OR "Alzheimer's dementia" OR "AD" OR "mild cognitive impairment" OR "huntington disease" OR "lewy body" OR "chronic cerebrovascular" OR "parkinson disease dementia" OR "parkinson dementia" OR “lewy bodies” OR “lewy neurites” OR “dementia, vascular” OR “vascular dementia*”)

#4	#1 OR #2 OR #3
#5	**(DE "Nursing") OR (DE "Caregiving") OR (DE "Long Term Care") OR (DE "Management")**
#6	**TI** (nursing OR "practical nursing" OR "care, nursing" OR "management, nursing Care" OR "care, long-term" OR "long term care" OR care OR caring OR caregiving OR cargiving OR manag* OR "long-term care")
#7	**AB** (nursing OR "practical nursing" OR "care, nursing" OR "management, nursing Care" OR "care, long-term" OR "long term care" OR care OR caring OR caregiving OR manag* OR "long-term care")
#8	#5 OR #6 OR #7
#9	**(DE "Nursing Homes") OR (DE "Residential Care Institutions")**
#10	**TI** ("elderly care institution" OR "nursing home*" OR "health care setting" OR "long term care facilities" OR "residential aged care" OR "care home*" OR "residential facilities" OR "homes for aged" OR "residential home*" OR "residential facilit*" OR "nursing home facility*" OR "nursing home care" OR "nursing home placement" OR "old people's home*" OR "homes for the aged" OR "old age home" OR "old age homes" OR "housing for the elderly")
#11	**AB** ("elderly care institution" OR "nursing home*" OR "health care setting" OR "long term care facilities" OR "residential aged care" OR "care home*" OR "residential facilities" OR "homes for aged" OR "residential home*" OR "residential facilit*" OR "nursing home facility*" OR "nursing home care" OR "nursing home placement" OR "old people's home*" OR "homes for the aged" OR "old age home" OR "old age homes" OR "housing for the elderly")
#12	#9 OR #10 OR #11
#13	**DE "Focus Group" OR DE "Focus Group Interview" OR DE "Qualitative Methods" OR DE "Grounded Theory" OR DE "Interpretative Phenomenological Analysis" OR DE "Narrative Analysis" OR DE "Semi-Structured Interview" OR DE "Thematic Analysis"**
#14	**TI** ("nursing methodology" OR "case study" OR "constant comparison" OR "content analysis" OR "descriptive study" OR "discourse analysis" OR ethnography OR exploratory OR feminist OR "focus group*" OR "grounded theory" OR hermeneutic OR interview OR narrative OR naturalistic OR "participant observation" OR phenomenology OR "qualitative method" OR "qualitative research" OR "qualitative study" OR "thematic analysis" OR "mixed method research" OR "mixed methods research")
#15	**AB** ("nursing methodology" OR "case study" OR "constant comparison" OR "content analysis" OR "descriptive study" OR "discourse analysis" OR ethnography OR exploratory OR feminist OR "focus group*" OR "grounded theory" OR hermeneutic OR interview OR narrative OR naturalistic OR "participant observation" OR phenomenology OR "qualitative method" OR "qualitative research" OR "qualitative study" OR "thematic analysis" OR "mixed method research" OR "mixed methods research")
#16	#13 OR #14 OR #15
#17	#4 AND #8 AND #12 AND #16

1. Search strategy for Wan Fang	
#1	**主题**:("痴呆" OR "老年痴呆" OR "血管性痴呆" OR "额颞叶性痴呆" OR "帕金森痴呆" OR "失智症" OR "认知症" OR "认知障碍" OR "阿尔茨海默" OR "阿尔兹海默" OR "Alzheimer" OR "AD" OR "轻度认知障碍" OR "MCI" OR "路易体痴呆")
#2	**主题**:("照护" OR "照顾" OR "护理" OR "管理" OR "照料" OR "照管" OR "照看" OR "看护")
#3	**主题**:("养老机构" OR "养老院" OR "护理之家" OR "幸福院" OR "老人院" OR "长期照护机构" OR "长期照顾机构" OR "长期护理机构" OR "长期看护机构" OR "敬老院" OR "安老院" OR "护理院" OR "老年人公寓")
#4	**主题**:("质性研究" OR "定性研究" OR "描述性研究" OR "民族志" OR "现象学" OR "扎根理论" OR "内容分析" OR "主题分析" OR "焦点小组" OR "解释学" OR "访谈" OR "叙述" OR "混合性研究")
#5	#1 AND #2 AND #3 AND #4
2. Search strategy for CNKI	
#1	**SU** %= ’痴呆’ + ’老年痴呆’ + ’血管性痴呆’ + ’额颞叶性痴呆’ + ’帕金森痴呆’ + ’失智症’ + ’认知症’ + ’认知障碍’ + ’阿尔茨海默’ + ’阿尔兹海默’ + ’Alzheimer’ + ’AD’ + ’轻度认知障碍’ + ’MCI’ + ’路易体痴呆’
#2	**SU** %= ’照护’ + ’照顾’ + ’护理’ + ’管理’ + ’照料’ + ’照管’ + ’照看’ + ’看护’
#3	**SU** %= ’养老机构’ + ’养老院’ + ’护理之家’ + ’幸福院’ + ’老人院’ + ’长期照护机构’ + ’长期照顾机构’ + ’长期护理机构’ + ’长期看护机构’ + ’敬老院’ + ’安老院’ + ’护理院’ + ’老年人公寓’
#4	**SU** %= ’质性研究’ + ’定性研究’ + ’描述性研究’ + ’民族志’ + ’现象学’ + ’扎根理论’ + ’内容分析’ + ’主题分析’ + ’焦点小组’ + ’解释学’ + ’访谈’ + ’叙述’ + ’混合型研究’
#5	#1 AND #2 AND #3 AND #4
3. Search strategy for CBM	
#1	**"痴呆, 血管性"[不加权:扩展] OR "阿尔茨海默病"[不加权:扩展] OR "Lewy 体病"[不加权:扩展] OR "阿尔茨海默病"[不加权:扩展] OR "认知障碍"[不加权:扩展]**
#2	"痴呆"**[摘要:智能]** OR "老年痴呆"[摘要:智能] OR "血管性痴呆"[摘要:智能] OR "额颞性痴呆"[摘要:智能] OR "帕金森痴呆"[摘要:智能] OR "失智症"[摘要:智能] OR "认知症"[摘要:智能] OR "认知障碍"[摘要:智能] OR "阿尔茨海默"[摘要:智能] OR "阿尔兹海默"[摘要:智能] OR "Alzheimer"[摘要:智能] OR "AD"[摘要:智能] OR "轻度认知障碍"[摘要:智能] OR "MCI"[摘要:智能] OR "路易体痴呆"[摘要:智能]
#3	"痴呆"**[标题:智能]** OR "老年痴呆"[标题:智能] OR "血管性痴呆"[标题:智能] OR "额颞性痴呆"[标题:智能] OR "帕金森痴呆"[标题:智能] OR "失智症"[标题:智能] OR "认知症"[标题:智能] OR "认知障碍"[标题:智能] OR "阿尔茨海默"[标题:智能] OR "阿尔兹海默"[标题:智能] OR "Alzheimer"[标题:智能] OR "AD"[标题:智能] OR "轻度认知障碍"[标题:智能] OR "MCI"[标题:智能] OR "路易体痴呆"[标题:智能]
#4	#1 OR #2 OR #3

For peer review only - http://bmjopen.bmj.com/site/about/guidelines.xhtml

#5	**″护理″[不加权:扩展]**
#6	″照护″**［摘要:智能］** OR ″照顾″［摘要:智能］ OR ″护理″［摘要:智能］ OR ″管理″［摘要:智能］ OR ″照料″［摘要:智能］ OR ″照管″［摘要:智能］ OR ″照看″［摘要:智能］ OR ″看护″［摘要:智能］
#7	″照护″**［标题:智能］** OR ″照顾″［标题:智能］ OR ″护理″［标题:智能］ OR ″管理″［标题:智能］ OR ″照料″［标题:智能］ OR ″照管″［标题:智能］ OR ″照看″［标题:智能］ OR ″看护″［标题:智能］
#8	#5 OR #6 OR #7
#9	**″老人疗养院″[不加权:扩展]**
#10	″养老机构″**［摘要:智能］** OR ″养老院″［摘要:智能］ OR ″护理之家″［摘要:智能］ OR ″幸福院″［摘要:智能］ OR ″老人院″［摘要:智能］ OR ″长期照护机构″［摘要:智能］ OR ″长期护理机构″［摘要:智能］ OR ″长期照顾机构″［摘要:智能］ OR ″长期看护机构″［摘要:智能］ OR ″敬老院″［摘要:智能］ OR ″安老院″［摘要:智能］ OR ″护理院″［摘要:智能］ OR ″老年人公寓″［摘要:智能］
#11	″养老机构″**［标题:智能］** OR ″养老院″［标题:智能］ OR ″护理之家″［标题:智能］ OR ″幸福院″［标题:智能］ OR ″老人院″［标题:智能］ OR ″长期照护机构″［标题:智能］ OR ″长期护理机构″［标题:智能］ OR ″长期照顾机构″［标题:智能］ OR ″长期看护机构″［标题:智能］ OR ″敬老院″［标题:智能］ OR ″安老院″［标题:智能］ OR ″护理院″［标题:智能］ OR ″老年人公寓″［标题:智能］
#12	#9 OR #10 OR #11
#13	″质性研究″**［摘要:智能］** OR ″定性研究″［摘要:智能］ OR ″描述性研究″［摘要:智能］ OR ″民族志″［摘要:智能］ OR ″现象学″［摘要:智能］ OR ″扎根理论″［摘要:智能］ OR ″内容分析″［摘要:智能］ OR ″主题分析″［摘要:智能］ OR ″焦点小组″［摘要:智能］ OR ″解释学″［摘要:智能］ OR ″访谈″［摘要:智能］ OR ″叙述″［摘要:智能］ OR ″混合型研究″［摘要:智能］
#14	″质性研究″**［标题:智能］** OR ″定性研究″［标题:智能］ OR ″描述性研究″［标题:智能］ OR ″民族志″［标题:智能］ OR ″现象学″［标题:智能］ OR ″扎根理论″［标题:智能］ OR ″内容分析″［标题:智能］ OR ″主题分析″［标题:智能］ OR ″焦点小组″［标题:智能］ OR ″解释学″［标题:智能］ OR ″访谈″［标题:智能］ OR ″叙述″［标题:智能］ OR ″混合型研究″［标题:智能］
#15	#13 OR #14
#16	#4 AND #8 AND #12 AND #15
4. Search strategy for VIP	
#1	**M**=(″痴呆″ + ″老年痴呆″ + ″失智症″ + ″认知症″ + ″认知障碍″ + ″阿尔茨海默″ + ″阿尔兹海默″ + ″AD″ + ″轻度认知障碍″ + ″MCI″ + ″额颞叶性痴″ + ″血管性痴呆″ + ″帕金森痴呆″ + ″Alzheimer″ + ″路易体痴呆″) OR **R**=(″痴呆″ + ″老年痴呆″ + ″失智症″ + ″认知症″ + ″认知障碍″ + ″阿尔茨海默″ + ″阿尔兹海默″ + ″AD″ + ″轻度认知障碍″ + ″MCI″ + ″额颞叶性痴″ + ″血管性痴呆″ + ″帕金森痴呆″ + ″Alzheimer″ + ″路易体痴呆″)
#2	**M**=(″照护″ + ″护理″ + ″照顾″ + ″照料″ + ″照看″ + ″照管″ + ″看护″) OR **R**=(″照护″ + ″护理″ + ″照顾″ + ″照料″ + ″照看″ + ″照管″ + ″看护″)
#3	**M**=(″养老机构″ + ″养老院″ + ″护理之家″ + ″幸福院″ + ″老人院″ + ″敬老院″ + ″安老院″ + ″护理院″ + ″老年人公寓″) OR **R**=(″养老机构″ + ″养老院″ + ″护理之家″ + ″幸福院″ + ″老人院″ + ″敬老院″ + ″安老院″ + ″护理院″ + ″老年人

For peer review only

	公寓”)
#4	**M**=(″质性研究″ + ″定性研究″ + ″描述性研究″ + ″民族志″ + ″现象学″ + ″扎根理论″ + ″内容分析″ + ″主题分析″ + ″焦点小组″ + ″解释学″ + ″访谈″ + ″叙述″ + ″混合性研究”) OR **R**=(″质性研究″ + ″定性研究″ + ″描述性研究″ + ″民族志″ + ″现象学″ + ″扎根理论″ + ″内容分析″ + ″主题分析″ + ″焦点小组″ + ″解释学″ + ″访谈″ + ″叙述″ + ″混合性研究”)
#5	#1 AND #2 AND #3 AND #4

For peer review only

JBI Critical Appraisal Checklist for Qualitative Research

Reviewer_____________________________________Date____________________________

Author _______________________________________Year_________Record Number______

	Yes	No	Unclear	Not applicable
1. Is there congruity between the stated philosophical perspective and the research methodology?	☐	☐	☐	☐
2. Is there congruity between the research methodology and the research question or objectives?	☐	☐	☐	☐
3. Is there congruity between the research methodology and the methods used to collect data?	☐	☐	☐	☐
4. Is there congruity between the research methodology and the representation and analysis of data?	☐	☐	☐	☐
5. Is there congruity between the research methodology and the interpretation of results?	☐	☐	☐	☐
6. Is there a statement locating the researcher culturally or theoretically?	☐	☐	☐	☐
7. Is the influence of the researcher on the research, and vice- versa, addressed?	☐	☐	☐	☐
8. Are participants, and their voices, adequately represented?	☐	☐	☐	☐
9. Is the research ethical according to current criteria or, for recent studies, and is there evidence of ethical approval by an appropriate body?	☐	☐	☐	☐
10. Do the conclusions drawn in the research report flow from the analysis, or interpretation, of the data?	☐	☐	☐	☐

Overall appraisal: Include ☐ Exclude ☐ Seek further info ☐

Comments (Including reason for exclusion)

JBI QARI Data Extraction Tool for Qualitative Research

Reviewer ______________________ Date ______________________

Author ______________________ Year ______________________

Journal ______________________ Record Number ______________________

Study Description

Methodology

Method

Phenomena of interest

Setting

Geographical

Cultural

Participants

Data analysis

Authors conclusions

Comments

Complete Yes ☐ No ☐

Copyright © The Jo0anna Briggs Institute 2014

Findings	Illustration form Publication (page number)	Evidence		
		Unequivocal	Credible	Unsupported

Extraction of findings complete Yes ☐ No ☐

Copyright © The Jo0anna Briggs Institute 2014

only

Reporting checklist for protocol of a systematic review and meta analysis.

Based on the PRISMA-P guidelines.

Instructions to authors

Complete this checklist by entering the page numbers from your manuscript where readers will find each of the items listed below.

Your article may not currently address all the items on the checklist. Please modify your text to include the missing information. If you are certain that an item does not apply, please write "n/a" and provide a short explanation.

Upload your completed checklist as an extra file when you submit to a journal.

In your methods section, say that you used the PRISMA-Preporting guidelines, and cite them as:

Moher D, Shamseer L, Clarke M, Ghersi D, Liberati A, Petticrew M, Shekelle P, Stewart LA. Preferred Reporting Items for Systematic Review and Meta-Analysis Protocols (PRISMA-P) 2015 statement. Syst Rev. 2015;4(1):1.

		Reporting Item	Page Number
**Title**			
Identification	#1a	Identify the report as a protocol of a systematic review	Lines 1-2
Update	#1b	If the protocol is for an update of a previous systematic review, identify as such	n/a

For peer review only

**Registration**			
	#2	If registered, provide the name of the registry (such as PROSPERO) and registration number	Lines 58-60
**Authors**			
Contact	#3a	Provide name, institutional affiliation, e-mail address of all protocol authors; provide physical mailing address of corresponding author	Lines 4-19
Contribution	#3b	Describe contributions of protocol authors and identify the guarantor of the review	Lines 282-288
**Amendments**			
	#4	If the protocol represents an amendment of a previously completed or published protocol, identify as such and list changes; otherwise, state plan for documenting important protocol amendments	n/a
**Support**			
Sources	#5a	Indicate sources of financial or other support for the review	Lines 280-281
Sponsor	#5b	Provide name for the review funder and / or sponsor	n/a
Role of sponsor or funder	#5c	Describe roles of funder(s), sponsor(s), and / or institution(s), if any, in developing the protocol	n/a
**Introduction**			

For peer review only

Rationale	#6	Describe the rationale for the review in the context of what is already known	Lines 74-139
Objectives	#7	Provide an explicit statement of the question(s) the review will address with reference to participants, interventions, comparators, and outcomes (PICO)	Lines 140-146
**Methods**			
Eligibility criteria	#8	Specify the study characteristics (such as PICO, study design, setting, time frame) and report characteristics (such as years considered, language, publication status) to be used as criteria for eligibility for the review	Lines 156-173
Information sources	#9	Describe all intended information sources (such as electronic databases, contact with study authors, trial registers or other grey literature sources) with planned dates of coverage	Lines 174-180
Search strategy	#10	Present draft of search strategy to be used for at least one electronic database, including planned limits, such that it could be repeated	Supplementary File 1
Study records - data management	#11a	Describe the mechanism(s) that will be used to manage records and data throughout the review	Lines 197-201
Study records - selection process	#11b	State the process that will be used for selecting studies (such as two independent reviewers) through each phase of the review (that is, screening, eligibility and	Lines 196-224

For peer review only

		inclusion in meta-analysis)	
Study records - data collection process	#11c	Describe planned method of extracting data from reports (such as piloting forms, done independently, in duplicate), any processes for obtaining and confirming data from investigators	Lines 225-241
Data items	#12	List and define all variables for which data will be sought (such as PICO items, funding sources), any pre-planned data assumptions and simplifications	Lines 228-233
Outcomes and prioritization	#13	List and define all outcomes for which data will be sought, including prioritization of main and additional outcomes, with rationale	Lines 161-164
Risk of bias in individual studies	#14	Describe anticipated methods for assessing risk of bias of individual studies, including whether this will be done at the outcome or study level, or both; state how this information will be used in data synthesis	Lines 212-224
Data synthesis	#15a	Describe criteria under which study data will be quantitatively synthesised	n/a
Data synthesis	#15b	If data are appropriate for quantitative synthesis, describe planned summary measures, methods of handling data and methods of combining data from studies, including any planned exploration of consistency (such as I2, Kendall's τ)	n/a
Data synthesis	#15c	Describe any proposed additional analyses (such as	n/a

For peer review only

		sensitivity or subgroup analyses, meta-regression)	
Data synthesis	#15d	If quantitative synthesis is not appropriate, describe the type of summary planned	Lines 242-260
Meta-bias(es)	#16	Specify any planned assessment of meta-bias(es) (such as publication bias across studies, selective reporting within studies)	Lines 71-72
Confidence in cumulative evidence	#17	Describe how the strength of the body of evidence will be assessed (such as GRADE)	Lines 261-270

None The PRISMA-P elaboration and explanation paper is distributed under the terms of the Creative Commons Attribution License CC-BY. This checklist can be completed online using https://www.goodreports.org/, a tool made by the EQUATOR Network in collaboration with Penelope.ai

For peer review only

BMJ Open

Barriers and facilitators to dementia care in long-term care facilities: Protocol for a qualitative systematic review and meta-synthesis

Journal:	*BMJ Open*
Manuscript ID	bmjopen-2023-076058.R2
Article Type:	Protocol
Date Submitted by the Author:	02-Oct-2023
Complete List of Authors:	Zhang, Xi; Xi'an Jiaotong University<br>Guan, Chengguo; Xi'an Jiaotong University<br>He, Jinjie; Xi'an Jiaotong University<br>Wang, Jing; Xian Jiaotong University
**Primary Subject Heading**:	Nursing
Secondary Subject Heading:	Geriatric medicine
Keywords:	Systematic Review, Dementia < NEUROLOGY, QUALITATIVE RESEARCH, Protocols & guidelines < HEALTH SERVICES ADMINISTRATION & MANAGEMENT

SCHOLARONE™
Manuscripts

Barriers and facilitators to dementia care in long-term care facilities：Protocol for a qualitative systematic review and meta-synthesis

Authors and affiliation

Xi Zhang[1], Chengguo Guan[1], Jinjie He[1], Jing Wang[1]*

[1]Health Science Center, Xi'an Jiaotong University, Xi'an, Shaanxi, China

Email

Xi Zhang: zhangxi1234560814@163.com

Chengguo Guan: novogcg@stu.xjtu.edu.cn

Jinjie He: hjj521209@stu.xjtu.edu.cn

Jing Wang*: novowj@xjtu.edu.cn

***Corresponding author**

Jing Wang

Health Science Center, Xi'an Jiaotong University

No.76 Yanta West Road, Xi'an, Shaanxi, China

Phone: +86-18789400330

Email: novowj@xjtu.edu.cn

Keywords:

dementia, long-term care facilities, qualitative study, systematic review, protocol

Word count

2366

For peer review only

ABSTRACT

Introduction: Long-term care needs for people with dementia are predicted to increase due to increased life expectancy and dementia diagnoses. Most published meta-synthesis of dementia care focus on hospital or home settings. When focusing on long-term care facilities, most reviews about dementia care only focus on a single outcome, such as feeding, behavioral symptoms management, palliative care, and others, which is limited. The present study aims to synthesise qualitative data and examine barriers and facilitators to caring for people with dementia in long-term care facilities.

Methods and analysis: This is the protocol for our systematic review and meta-synthesis, which describes the design of this study, and we plan to complete the study from October 2023 to November 2024. The systematic review and meta-synthesis will follow the Joanna Briggs Institute (JBI) guidance for systematic reviews of qualitative evidence. Nine databases (five English and four Chinese) were searched, including Embase, Web of Science, Medline, CINAHL, PsycINFO and Wan Fang Data, China National Knowledge Infrastructure (CNKI), VIP, and Chinese Biomedical Medicine (CBM), from inception to August 2023. Qualitative and mixed-approach researches about barriers and facilitators to caring for people with dementia in long-term care facilities, which are reported in English or Chinese, will be included. Covidence software will help with study selection, assessment, and data extraction. The JBI Critical Appraisal Checklist for Qualitative Research (2020) will be used for included studies' quality assessment. Data extraction will be based on the JBI-QARI Data Extraction Tool for Qualitative Research. The JBI aggregation approach will be used to synthesises data. We will use the JBI ConQual tool to assess the credibility and dependability of each synthesised finding to establish confidence in the synthesized findings. All review steps will be managed by two reviewers independently, disparities will be discussed. If consensus cannot reach a resolution, a third reviewer will be consulted.

Ethics and dissemination: The present study is a secondary analysis of published qualitative data. So ethical approval is not required. The findings may be disseminated

For peer review only

through peer-reviewed publications, conference papers or elsewhere.

Registration details: The protocol was registered with the International Prospective Register of Systematic Reviews (PROSPERO) in May 2022, and the registration number is CRD42022326178.

Keywords: dementia, long-term care facilities, qualitative study, systematic review, protocol

ARTICLE SUMMARY

Strengths and limitations of this study

- This study will be guided by the Joanna Briggs Institute methodology for qualitative systematic reviews to increase the accuracy and reliability of results.
- The consensus-based approach to analysis will ensure shared responsibility for interpretative decisions and the rigor and trustworthiness of the findings of this study.
- A limitation is the exclusion of studies not written in English or Chinese, and of grey literature.

INTRODUCTION

Dementia is a degenerative syndrome affecting individuals' cognitive functions. It impacts memory, behavior, thinking, and social abilities as the disease advances. Those affected become increasingly dependent on care from others for all activities of daily living.[1] Previous studies show that age is a critical factor in dementia, and there is no cure for dementia currently.[2] The demographic shift to an aging population has rapidly increased the number of people with dementia. According to the most recent World Health Organization (WHO) figures, 55.2 million people worldwide were living with dementia in 2019, and the number is expected to rise to 139 million in 2050.[3] Caring for people with dementia requires round-the-clock care, and caregivers must manage behavioral and psychological symptoms (such as aggression, wandering, and depression), placing an overwhelming burden on the caregivers' well-being, especially

For peer review only

in the severe stage of dementia.[4, 5] Long-term care facilities are the alternative place when people with dementia cannot get sufficient support at home.[6, 7] Long-term care facilities may have different names in different countries, like nursing homes, skilled nursing facilities, assisted living facilities, and residential facilities, providing services to people who cannot live independently.[8] Approximately over 30% of long-term care facility residents have a form of dementia in higher-income economies.[3] In lower- and middle-income countries, informal caregivers, mostly family members, provide a significant proportion of dementia care nowadays.[3] However, long-term care needs for people with dementia are predicted to increase due to increased life expectancy and dementia diagnoses.

The WHO Framework for Countries to achieve an integrated continuum of long-term care recognizes critical aspects for an integrated continuum service provision in long-term care facilities and guides countries in evaluating and implementing equitable and sustainable care in long-term care facilities.[9] The goal of long-term care is to ensure that an individual who has significant declines in physical or mental capacity can maintain the best possible quality of life, with the greatest possible degree of independence, autonomy, participation, personal fulfillment and human dignity.[9] Professionals need to take a holistic approach to providing care to the people living with dementia. Depending on the situation, dementia care should include basic care such as eating and dressing, pain management, behavioral and psychological symptoms management, and palliative care to promote well-being and quality of life.[10] However, knowledge-practice gaps about dementia in long-term care facilities have been revealed by several studies, such as quality of life still needing to be improved,[11] while feeding difficulties,[12] pain management,[13] and other disease-related care needs require further attention. Besides, behavioral and psychological symptoms are common in people with dementia, and how to manage these symptoms is still an endeavor.[14] Professionals, as key service providers in long-term care facilities, face many difficulties in providing care for people with dementia.[15-18] Some qualitative studies suggest that professionals find it hard to provide personalized care and interact with people in long-term care facilities because of insufficient time and a lack of knowledge

For peer review only

about dementia.[15, 17] It has also been noted that when a professional's attitude towards dementia is positive and hopeful, the more it contributes to the caring of people with dementia.[19] In addition to exploring the experiences of professionals in holistic dementia care, there are also studies that focus on a single outcome. Some studies suggest that management of behavioral and psychological symptoms may be limited by lack of clinical guidance and resources,[20] and feeding care is also associated with clinical resources, in addition to the environment.[21]

In recent years, qualitative studies are proliferating in nursing research because they effectively complement quantitative research results.[22] Qualitative studies explore detailed explanations and meanings shown by participants, providing an alternative perspective to a more positivist approach.[23] Meta-synthesis of qualitative studies can yield broader results because they synthesize a more comprehensive range of participants and descriptions. It integrates the body of knowledge about a topic and, as a result of this process, can produce new interpretations of the phenomenon that contribute to understanding the topic.[24] To date, a limited number of qualitative studies have explored the experience of dementia care from the perspective of professionals. There has been some meta-analysis based on these articles, but most published meta-synthesis of dementia care focus on hospital[25-27] or home settings.[28-30] When focusing on long-term care facilities, most reviews about dementia care only focus on a single outcome, such as feeding,[31, 32] behavioral symptoms management,[33, 34] palliative care,[35] and others, which is limited. The dementia care is holistic but considering only one aspect of care is not comprehensive. Integrating these known factors to identify barriers and facilitators to dementia care in long-term care facilities is essential.

To the best of our knowledge, there are currently no studies that have systematically evaluated qualitative research on holistic dementia care in long-term care facilities. Therefore, the present study aims to systematically review and synthesise the best available qualitative evidence on barriers and facilitators to caring for people with dementia in long-term care facilities from the perspective of the professionals, providing more comprehensive evidence for better service development in dementia

For peer review only - http://bmjopen.bmj.com/site/about/guidelines.xhtml

care in long-term care facilities.

METHOD AND ANALYSIS

Design

The protocol was registered with the International Prospective Register of Systematic Reviews (PROSPERO) in May 2022, and the registration number is CRD42022326178. This protocol was designed following the Preferred reporting items for systematic review and meta-analysis protocols (PRISMA-P) 2015 statement[36, 37] and the Joanna Briggs Institute (JBI) guidance for systematic reviews of qualitative evidence.[38] The study will run from October 2023 to November 2024.

Eligibility criteria

Type of participants: Studies focusing on health professionals (aged≥18) caring for people with dementia in long-term care facilities, including doctors, nurses, and other health professionals. Studies focusing on multiple disease types (not only care for people with dementia) will also be excluded.

Phenomena of interest: Studies focusing on barriers and facilitators to caring for people with dementia in long-term care facilities, including experiences in basic care such as eating and dressing, pain management, behavioral and psychological symptoms management, palliative care and other related care.

Context: Studies focusing on long-term care facilities,[8] such as nursing homes, skilled nursing facilities, assisted living facilities, nursing institutes, and residential facilities, providing services to people who cannot live independently. Studies conducted in adult daycare facilities, respite care, and other transitional care facilities will also be included.

Types of studies: Qualitative and mixed methods studies with an analytical part of the qualitative content published in English or Chinese will be included. Only the qualitative component of the mixed methods studies will be considered. Quantitative studies, pilot studies, reviews, case reports, case series, reanalysis of data, conference abstracts, and expert opinions will be excluded.

Information sources

For peer review only

Nine databases will be searched, including five English databases and four Chinese databases with no time limit applied: The five English databases are Embase, Web of Science, Medline, CINAHL, PsycINFO and the four Chinese databases are Wan Fang Data, China National Knowledge Infrastructure (CNKI), VIP and Chinese Biomedical Medicine (CBM). References and citations of the included studies will also be searched to identify if there are any other relevant sources.

Search strategy

Keywords were first formed based on the eligibility criteria, and synonyms of each keyword were then analysed to create a logic grid to capture relevant studies. Boolean operators will be used to combine all the keywords. Pilot searches will be conducted to confirm the sensitivity and specificity of the strategy in all databases before the formal search is conducted. The final strategy will be consulted by an expert librarian and reviewed by all team members. The index terms and the search strategy were developed in Web of Science among five English databases and China National Knowledge Infrastructure among four Chinese databases. The Web of Science English search strategy and China National Knowledge Infrastructure Chinese search strategy will be then translated and used for additional databases. We will include studies retrievable from the establishment of the database to August 2023. Search strategies are available in Supplement 1. Citation tracing will also be conducted, with the reference lists of included studies being screened to identify studies that were missed in the search process.

Study selection

All search records will be imported to Endnote X9 (V.9.3.3) for duplicates removal and then to Covidence software (https://app.covidence.org/reviews/active) for study selection. Covidence is a systematic review production tool developed by researchers specialized in the systematic review process to standardize the process of conducting systematic reviews. According to the proposed eligibility criteria, title and abstract screening will be conducted by two reviewers (CG and XZ) independently on all records. Then, the full-text review of included studies will be screened in the same way, and the reason for exclusion will be selected and recorded in Covidence. Before each

For peer review only

formal process, a pilot test will be conducted initially with 5-10 percent of studies screened by all reviewers independently to facilitate understanding and application of eligibility criteria and the tool. Disparities will be discussed during the screening process via group discussion. If a resolution cannot be reached by consensus, the third and fourth reviewers (JH and JW) will be consulted. Regular group meetings with all the reviewers will be held regularly to discuss and resolve any disagreements. The results of the screening will be recorded in a PRISMA flow chart.

Risk of bias assessment

The JBI Critical Appraisal Checklist for Qualitative Research (2020) (Supplement 2) will be used for assessing the quality of included studies, which includes ten items that assess the studies' philosophical foundation, methodology, method of data collection, method of data analysis, results interpretation, the logic of studies and research ethics. The four responses "yes," "no," "unclear," and "not applicable" will be used to evaluate each item. The final quality rating of the study will be rated as high, medium, or low based on the assessment of individual items and the amount of weight given to the degree to which each item impacted the findings of the study.[38, 39] Only studies having at least a medium ranking will be included. Two reviewers (CG and XZ) will appraise all included studies' methodological quality independently and disparities will be discussed. The third and fourth reviewers (JH and JW) will be consulted when a resolution cannot be reached by consensus.

For peer review only

Data extraction

Data extraction will be conducted based on the JBI Qualitative Assessment and Review Instrument (JBI-QARI) Data Extraction Tool for Qualitative Research (Supplement 3) by two reviewers (CG and XZ) independently.[38] According to the tool, the characteristics of included studies and findings with illustrations will be extracted. Information will be extracted using Covidence, including (1) author, publication, year, country, geographical location, and study setting; (2) participants' characteristics and sample size; (3) research design, methodology, and methods; (4) phenomena of interest and main findings. Before the commencement of the formal data extraction, data from 5-10 percent of the included studies will be extracted using the data extraction tool to

pilot test the tool and minimize the risk of error. According to the JBI review manual, all the findings extracted from the selected studies will be evaluated and divided into three levels based on the degree of support between the finding and its illustration: unequivocal, credible and unsupported.[38] Only the findings rated as unequivocal or credible will be included for data synthesis. Any disagreements between the two reviewers will be discussed and resolved by regular team meetings with the four reviewers.

Data synthesis

The JBI meta aggregation approach with a three-step process will be used for data synthesis after data extraction.[40] In the three-step data synthesis process, the findings will first be extracted from the original studies, then the included findings will be grouped into categories based on similarity of meaning, and similar categories will ultimately be further synthesized into different synthesized findings based on their relationships and relevance to the research objective of this review. This approach focuses on the essence of qualitative research and emphasizes the value and role of qualitative research in an evidence-based healthcare service system, collecting all included studies' findings and summarizing them according to their meanings to make them more targeted, convincing, and generalizing. On the premise of understanding the philosophical foundation and methodology of various qualitative studies, reviewers repeatedly read, understand, analyze, and interpret the meaning of each included findings, combine similar findings, form a new category, and finally summarize the category as synthesized findings. In the present review, the findings of each included study will be read carefully to understand completely before data synthesis. Regular meetings will be held with the four reviewers to reach an agreement on the final synthesized findings, and any conflicting opinions between the reviewers will be settled by thorough discussions.

Assessing the confidence of findings

We will use the JBI ConQual tool to assess the confidence of each synthesised finding.[41] Dependability and credibility are two elements to influence the confidence of qualitative synthesised findings. Dependability can be established by examining the

For peer review only

quality of the original studies included through a set of critical appraisal questions. Credibility evaluates the goodness of fit between the author's interpretation and the original data. First, the confidence of synthesised findings will be initially assumed to be graded as high, and then downgraded based on the results of dependability and credibility assessments, and finally each synthesised finding will be rated high, moderate, low, or very low.[41]

Patient and public involvement

None.

Ethics and dissemination

The present study is a secondary analysis of published qualitative data; there is no concern for privacy. So ethical approval is not required for the present study. However, the research ethics of each included original study will be assessed rigorously. The findings may be disseminated through peer-reviewed publications, conference papers or elsewhere.

Acknowledgments

We gratitude for the help and support of the National Social Science Fund of China.

Author Contributions

JW and CG conceived the topic of this study. All team members (JW, CG, JH, XZ) participated in the discussion of the study design and made significant comments. JW and CG constructed the search strategy. CG, JH, XZ, and JW will conduct data selection, assessment, extraction, and synthesis. XZ drafted the protocol manuscript, and then the manuscript was reviewed by JW. All authors approved the submitted protocol and are accountable for its content.

Funding statement

This work was supported by The National Social Science Fund of China (Grant No. 20XRK004).

Competing interests

none

For peer review only

References

1. World Alzheimer Report 2021: Journey through the diagnosis of dementia. *Alzheimer's Disease International,* 2021. https://www.alzint.org/resource/world-alzheimer-report-2021/.

2. Jia L, Du Y, Chu L, et al. Prevalence, risk factors, and management of dementia and mild cognitive impairment in adults aged 60 years or older in China: a cross-sectional study. *Lancet Public Health* 2020;5(12):e661-e71. doi: 10.1016/s2468-2667(20)3015-7

3. WHO Global status report on the public health response to dementia Executive summary. *World Health Organization (WHO),* 2021. https://www.who.int/multi-media/details/global-status-report-on-the-public-health-response-to-dementia.

4. Wang J, Xiao LD, Li X, et al. Caregiver distress and associated factors in dementia care in the community setting in China. *Geriatr Nurs* 2015;36(5):348-54. doi: 10.1016/j.gerinurse.2015.04.013

5. Connors MH, Seeher K, Teixeira-Pinto A, et al. Dementia and caregiver burden: A three-year longitudinal study. *Int J Geriatr Psychiatry* 2020;35(2):250-58. doi: 10.1002/gps.5244

6. Xiao LD, Wang J, He GP, et al. Family caregiver challenges in dementia care in Australia and China: a critical perspective. *BMC Geriatr* 2014;14:6. doi: 10.1186/1471-2318-14-6

7. Gaugler JE, Mittelman MS, Hepburn K, et al. Predictors of change in caregiver burden and depressive symptoms following nursing home admission. *Psychol Aging* 2009;24(2):385-96. doi: 10.1037/a0016052

8. Preventing and managing COVID-19 across long-term care services: Policy brief, 24 July 2020. *world Health Organization (WHO),* 2020. https://www.who.int/publications/i/item/WHO-2019-nCoV-Policy_Brief-Long-term_Care-2020.1.

9. Framework for countries to achieve an integrated continuum of long-term care. *World Health Organization (WHO)* , 2021. https://www.who.int/publications/i/item/9789240038844.

10. Fazio S, Pace D, Maslow K, et al. Alzheimer's Association Dementia Care Practice Recommendations. *Gerontologist* 2018;58(suppl_1):S1-s9. doi: 10.1093/geront/gnx182

11. Olsen C, Pedersen I, Bergland A, et al. Differences in quality of life in home-dwelling persons and nursing home residents with dementia - a cross-sectional study. *BMC Geriatr* 2016;16:137. doi: 10.1186/s12877-016-0312-4

12. Perry E, Walton K, Lambert K. Prevalence of Malnutrition in People with Dementia in Long-Term Care: A Systematic Review and Meta-Analysis. *Nutrients* 2023;15(13). doi: 10.3390/nu15132927

13. Cole CS, Carpenter JS, Chen CX, et al. Prevalence and Factors Associated with Pain in Nursing Home Residents: A Systematic Review of the Literature. *J Am Med Dir Assoc* 2022;23(12):1916-25.e1. doi: 10.1016/j.jamda.2022.08.008

14. O'Donnell E, Holland C, Swarbrick C. Strategies used by care home staff to manage behaviour that challenges in dementia: A systematic review of qualitative studies. *Int J Nurs Stud* 2022;133:104260. doi: 10.1016/j.ijnurstu.2022.104260

15. Barbosa A, Nolan M, Sousa L, et al. Dementia in Long-term Care Homes: Direct Care Workers' Difficulties. *Procedia - Social and Behavioral Sciences* 2014;140:172-77. org/10.1016/j.sbspro.2014.04.405

16. Zhao Y, Liu L, Ding Y, et al. Understanding dementia care in care home setting in China: An exploratory qualitative study. *Health Soc Care Community* 2021;29(5):1511-21. doi: 10.1111/hsc.13213

17. Kong EH, Kim H, Kim H. Nursing home staff's perceptions of barriers and needs in implementing person-centred care for people living with dementia: A qualitative study. *J Clin Nurs* 2022;31(13-14):1896-906. doi: 10.1111/jocn.15729

18. Law K, Patterson TG, Muers J. Experiences of healthcare assistants working with clients with dementia in residential care homes. *Dementia (London)* 2019;18(2):644-59. doi: 10.1177/1471301216688396

19. Gerritsen DL, van Beek APA, Woods RT. Relationship of care staff attitudes with social well-being and challenging behavior of nursing home residents with dementia: a cross sectional study. *Aging Ment Health* 2019;23(11):1517-23. doi:

For peer review only

10.1080/13607863.2018.1506737

20. Jennings AA, Foley T, McHugh S, et al. 'Working away in that Grey Area…' A qualitative exploration of the challenges general practitioners experience when managing behavioural and psychological symptoms of dementia. *Age Ageing* 2018;47(2):295-303. doi: 10.1093/ageing/afx175

21. Sadarangani T, Chong S, Park S, et al. A Qualitative Analysis of the Delivery of Person-Centered Nutrition to Asian Americans With Dementia in the Adult Day Health Care Setting. *J Appl Gerontol* 2021;40(2):179-88. doi: 10.1177/0733464820910030

22. Wang J, Li X, Liu W, et al. The positive aspects of caregiving in dementia: A scoping review and bibliometric analysis. *Front Public Health* 2022;10:985391. doi: 10.3389/fpubh.2022.985391

23. Lindgren BM, Lundman B, Graneheim UH. Abstraction and interpretation during the qualitative content analysis process. *Int J Nurs Stud* 2020;108:103632. doi: 10.1016/j.ijnurstu.2020.103632

24. Evans D, Pearson A. Systematic reviews of qualitative research. *Clinical Effectiveness in Nursing* 2005;5:111-19. doi: 10.1054/cein.2001.0219

25. Brossard Saxell T, Ingvert M, Lethin C. Facilitators for person-centred care of inpatients with dementia: A meta-synthesis of registered nurses' experiences. *Dementia (London)* 2021;20(1):188-212. doi: 10.1177/1471301219871408

26. Moonga J, Likupe G. A systematic literature review on nurses' and health care support workers' experiences of caring for people with dementia on orthopaedic wards. *J Clin Nurs* 2016;25(13-14):1789-804. doi: 10.1111/jocn.13158

27. Digby R, Lee S, Williams A. The experience of people with dementia and nurses in hospital: an integrative review. *J Clin Nurs* 2017;26(9-10):1152-71. doi: 10.1111/jocn.13429

28. Köhler K, Dreyer J, Hochgraeber I, et al. Towards a middle-range theory of 'Stability of home-based care arrangements for people living with dementia' (SoCA-Dem): findings from a meta-study on mixed research. *BMJ Open* 2021;11(4):e042515. doi: 10.1136/bmjopen-2020-042515

29. Dawson A, Bowes A, Kelly F, et al. Evidence of what works to support and sustain

For peer review only

care at home for people with dementia: a literature review with a systematic approach. *BMC Geriatr* 2015;15:59. doi: 10.1186/s12877-015-0053-9

30. Marulappa N, Anderson NN, Bethell J, et al. How to implement person-centred care and support for dementia in outpatient and home/community settings: Scoping review. *BMC Health Serv Res* 2022;22(1):541. doi: 10.1186/s12913-022-07875-w

31. Li Y, Zhang X, Su J, et al. Optimizing mealtime care for people with dementia from the perspective of formal caregivers: A systematic review of qualitative research. *Int J Nurs Stud* 2021;123:104046. doi: 10.1016/j.ijnurstu.2021.104046.

32. Faraday J, Salis C, Barrett A. Equipping Nurses and Care Staff to Manage Mealtime Difficulties in People With Dementia: A Systematic Scoping Review of Training Needs and Interventions. *Am J Speech Lang Pathol* 2019;28(2):717-42. doi: 10.1044/2018_ajslp-18-0062

33. Walsh KA, Dennehy R, Sinnott C, et al. Influences on Decision-Making Regarding Antipsychotic Prescribing in Nursing Home Residents With Dementia: A Systematic Review and Synthesis of Qualitative Evidence. *J Am Med Dir Assoc* 2017;18(10):897.e1-97.e12. doi: 10.1016/j.jamda.2017.06.032

34. Jennings AA, Foley T, Walsh KA, et al. General practitioners' knowledge, attitudes, and experiences of managing behavioural and psychological symptoms of dementia: A mixed-methods systematic review. *Int J Geriatr Psychiatry* 2018;33(9):1163-76. doi: 10.1002/gps.4918

35. van der Steen JT, Radbruch L, Hertogh CM, et al. White paper defining optimal palliative care in older people with dementia: a Delphi study and recommendations from the European Association for Palliative Care. *Palliat Med* 2014;28(3):197-209. doi: 10.1177/0269216313493685

36. Moher D, Shamseer L, Clarke M, et al. Preferred reporting items for systematic review and meta-analysis protocols (PRISMA-P) 2015 statement. *Syst Rev* 2015;4(1):1. doi: 10.1186/2046-4053-4-1

37. Shamseer L, Moher D, Clarke M, et al. Preferred reporting items for systematic review and meta-analysis protocols (PRISMA-P) 2015: elaboration and explanation. *Bmj* 2015;350:g7647. doi: 10.1136/bmj.g7647

For peer review only

38. Lockwood C, Porritt K, Munn Z, et al. Chapter 2: Systematic reviews of qualitative evidence. *JBI Manual for Evidence Synthesis*; JBI, 2020. https://synthesismanual.jbi.global.

39. Ying G, Huiwen Z, Yingfeng Z, et cl. JBI evidence-based health center's quality assessment tool for different types of research- The quality evaluation of systematic review. *Journal of Nurses Training*, 2018;33(08):701-703. doi:10.16821/j.cnki.hsjx.2018.08.008.

40. Lockwood C, Munn Z, Porritt K. Qualitative research synthesis: methodological guidance for systematic reviewers utilizing meta-aggregation. *Int J Evid Based Healthc* 2015;13(3):179-87. doi: 10.1097/xeb.0000000000000062

41. Munn Z, Porritt K, Lockwood C, et al. Establishing confidence in the output of qualitative research synthesis: the ConQual approach. *BMC Med Res Methodol* 2014;14:108. doi: 10.1186/1471-2288-14-108

For peer review only

Barriers and facilitators to dementia care in long-term care facilities：A systematic review and meta-synthesis protocol

Supplement file 1 - Searched databases and search strategies

1. Search strategy for Web of Science	
#1	**TI=**(dement* OR "presenile dementia" OR "cognitive impairment" OR "cognitive dysfunction" OR "Alzheimer's disease" OR "Alzheimer's dementia" OR AD OR "mild cognitive impairment" OR "huntington disease" OR "lewy body" OR "chronic cerebrovascular" OR "parkinson disease dementia" OR "parkinson dementia" OR “lewy bodies” OR “lewy neurites” OR “dementia, vascular” OR “vascular dementia*”) OR **AB=**(dementia OR "presenile dementia" OR "cognitive impairment" OR "cognitive dysfunction" OR "Alzheimer's disease" OR "Alzheimer's dementia" OR AD OR "mild cognitive impairment" OR "huntington disease" OR "lewy body" OR "chronic cerebrovascular" OR "parkinson disease dementia" OR "parkinson dementia" OR “lewy bodies” OR “lewy neurites” OR “dementia, vascular” OR “vascular dementia*”)
#2	**TI=**(nursing OR "practical nursing" OR "care, nursing" OR "management, nursing Care" OR "care, long-term" OR "long term care" OR care OR caring OR caregiving OR manag* OR "long-term care") OR **AB=**(nursing OR "practical nursing" OR "care, nursing" OR "management, nursing Care" OR "care, long-term" OR "long term care" OR care OR caring OR caregiving OR manag* OR "long-term care")
#3	**TI=**("elderly care institution" OR "nursing home*" OR "health care setting" OR "long term care facilities" OR "residential aged care" OR "care home*" OR "residential facilities" OR "homes for aged" OR "residential home*" OR "residential facilit*" OR "nursing home facility*" OR "nursing home care" OR "nursing home placement" OR "old people's home*" OR "homes for the aged" OR "old age home" OR "old age homes" OR "housing for the elderly") OR **AB=**("elderly care institution" OR "nursing home*" OR "health care setting" OR "long term care facilities" OR "residential aged care" OR "care home*" OR "residential facilities" OR "homes for aged" OR "residential home*" OR "residential facilit*" OR "nursing home facility*" OR "nursing home care" OR "nursing home placement" OR "old people's home*" OR "homes for the aged" OR "old age home" OR "old age homes" OR "housing for the elderly")
#4	**TI=**("nursing methodology" OR "case study" OR "constant comparison" OR "content analysis" OR "descriptive study" OR "discourse analysis" OR ethnography OR exploratory OR feminist OR "focus group*" OR "grounded theory" OR hermeneutic OR interview OR narrative OR naturalistic OR "participant observation" OR phenomenology OR "qualitative method" OR "qualitative research" OR "qualitative study" OR "thematic analysis" OR "mixed method research" OR "mixed methods research") OR **AB=**("nursing methodology" OR "case study" OR "constant comparison" OR "content analysis" OR "descriptive study" OR "discourse analysis" OR ethnography OR exploratory OR feminist OR "focus group*" OR "grounded theory" OR hermeneutic OR interview OR narrative OR naturalistic OR "participant observation" OR phenomenology OR "qualitative method" OR "qualitative research" OR

	"qualitative study" OR "thematic analysis" OR "mixed method research" OR "mixed methods research")
#5	#1 AND #2 AND #3 AND #4
2. Search strategy for Embase	
#1	**'Alzheimer disease'/exp OR 'dementia'/exp OR 'mild cognitive impairment'/exp OR 'Lewy body'/exp OR 'multiinfarct dementia'/exp**
#2	dement*:**ti,ab** OR 'presenile dementia':ti,ab OR 'cognitive impairment':ti,ab OR alzheimer*:ti,ab OR ad:ti,ab OR 'mild cognitive impairment':ti,ab OR 'huntington disease':ti,ab OR 'lewy body':ab OR 'chronic cerebrovascular':ti,ab OR 'parkinson disease dementia':ti,ab OR 'parkinson dementia':ti,ab OR 'cognitive dysfunction':ti,ab OR 'lewy bodies':ti,ab OR 'lewy neurites':ti,ab OR 'dementia, vascular':ti,ab OR 'multiinfarct dementia': ti,ab
#3	#1 OR #2
#4	**'management'/exp OR 'nursing'/exp OR 'practical nursing'/exp OR 'care'/exp OR 'custodial care'/exp OR 'nursing care'/exp OR 'long term care'/exp**
#5	nursings:**ti,ab** OR 'practical nursing':ti,ab OR 'nursing care':ti,ab OR 'management, nursing care':ti,ab OR 'care, long-term':ti,ab OR 'long term care':ti,ab OR care:ti,ab OR caring:ti,ab OR caregiving:ti,ab OR manag*:ti,ab OR 'long-term care':ti,ab
#6	#4 OR #5
#7	**'nursing home'/exp OR 'residential home'/exp OR 'home for the aged'/exp**
#8	'elderly care institution':**ti,ab** OR 'health care setting':ti,ab OR 'long term care facilities':ti,ab OR 'residential aged care':ti,ab OR 'care home*':ti,ab OR 'nursing home*':ti,ab OR 'residential facilities':ti,ab OR 'homes for aged':ti,ab OR 'residential home*':ti,ab OR 'residential facilit*':ti,ab OR 'nursing home facility*':ti,ab OR 'nursing home care':ti,ab OR 'nursing home placement':ti,ab OR 'homes for the aged':ti,ab OR 'old age home':ti,ab OR 'old age homes':ti,ab OR 'housing for the elderly':ti,ab
#9	#7 OR #8
#10	**'qualitative research'/exp**
#11	'nursing methodology':**ti,ab** OR 'case study':ti,ab OR 'constant comparison':ti,ab OR 'content analysis':ti,ab OR 'descriptive study':ti,ab OR 'discourse analysis':ti,ab OR ethnography:ti,ab OR exploratory:ti,ab OR feminist:ti,ab OR 'focus group*':ti,ab OR 'grounded theory':ti,ab OR hermeneutic:ti,ab OR interview:ti,ab OR narrative:ti,ab OR naturalistic:ti,ab OR 'participant observation':ti,ab OR phenomenology:ti,ab OR 'qualitative method':ti,ab OR 'qualitative research':ti,ab OR 'qualitative study':ti,ab OR 'thematic analysis':ti,ab OR 'mixed method research':ti,ab OR 'mixed methods research':ti,ab
#12	#10 OR #11
#13	#3 AND #6 AND #9 AND #12 AND
3. Search strategy for Medline	
#1	**exp Dementia/ or exp Frontotemporal Dementia/ or exp Dementia, Vascular/ or Cognitive Dysfunction/ or exp Lewy Bodies/ or exp Parkinson Disease/**
#2	(dement* or "presenile dementia" or "cognitive impairment" or "cognitive dysfunction" or "Alzheimer's disease" or "Alzheimer's dementia" or AD or "mild cognitive impairment" or "huntington disease" or "lewy body" or "chronic cerebrovascular" or "parkinson disease dementia" or "parkinson dementia" or "parkinson dementia").tw.

For peer review only

#3	#1 or #2
#4	**exp Nursing/ or exp Nursing, Practical/ or exp Custodial Care/ or Nursing Care/ or exp Long-Term Care/**
#5	(nursing or "practical nursing" or "care, nursing" or "management, nursing Care" or "care, long-term" or "long term care" or care or caring or caregiving or manag* or "long-term care").tw.
#6	#4 or #5
#7	**exp Nursing Homes/ or exp Residential Facilities/ or Homes for the Aged/**
#8	("elderly care institution" or "nursing home*" or "health care setting" or "long term care facilities" or "residential aged care" or "care home*" or "residential facilities" or "homes for aged" or "residential home*" or "residential facilit*" or "nursing home facility*" or "nursing home care" or "nursing home placement" or "old people's home*" or "homes for the aged" or "old age home" or "old age homes" or "housing for the elderly").tw.
#9	#7 or #8
#10	**exp Qualitative Research/**
#11	("nursing methodology" or "case study" or "constant comparison" or "content analysis" or "descriptive study" or "discourse analysis" or ethnography or exploratory or feminist or "focus group*" or "grounded theory" or hermeneutic or interview or narrative or naturalistic or "participant observation" or phenomenology or "qualitative method" or "qualitative research" or "qualitative study" or "thematic analysis" or "mixed method research" or "mixed methods research").tw.
#12	#10 or #11
#13	#3 and #6 and #9 and #12
4. Search strategy for CINAHL	
#1	**(MH "Dementia+") OR (MH "Frontotemporal Dementia+") OR (MH "Dementia, Vascular+") OR (MH "Lewy Body Disease") OR (MH "Mild Cognitive Impairment")**
#2	**TI** (dement* OR "presenile dementia" OR "cognitive impairment" OR "cognitive dysfunction" OR "Alzheimer's disease" OR "Alzheimer's dementia" OR AD OR "mild cognitive impairment" OR "huntington disease" OR "lewy body" OR "chronic cerebrovascular" OR "parkinson disease dementia" OR "parkinson dementia" OR “lewy bodies” OR “lewy neurites” OR “dementia, vascular” OR “vascular dementia*”)
#3	**AB** (dement* OR "presenile dementia" OR "cognitive impairment" OR "cognitive dysfunction" OR "Alzheimer's disease" OR "Alzheimer's dementia" OR AD OR "mild cognitive impairment" OR "huntington disease" OR "lewy body" OR "chronic cerebrovascular" OR "parkinson disease dementia" OR "parkinson dementia" OR “lewy bodies” OR “lewy neurites” OR “dementia, vascular” OR “vascular dementia*”)
#4	#1 OR #2 OR #3
#5	**(MH "Caring+") OR (MH "Long Term Care") OR (MH "Management+")**
#6	**TI** (nursing OR "practical nursing" OR "care, nursing" OR "management, nursing Care" OR "care, long-term" OR "long term care" OR care OR caring OR caregiving OR manag* OR "long-term care")
#7	**AB** (nursing OR "practical nursing" OR "care, nursing" OR "management, nursing Care" OR "care, long-term" OR "long term care" OR care OR caring OR caregiving OR manag* OR "long-term care")

#8	#5 OR #6 OR #7
#9	**(MH "Nursing Homes+") OR (MH "Residential Facilities+")**
#10	**TI** ("elderly care institution" OR "nursing home*" OR "health care setting" OR "long term care facilities" OR "residential aged care" OR "care home*" OR "residential facilities" OR "homes for aged" OR "residential home*" OR "residential facilit*" OR "nursing home facility*" OR "nursing home care" OR "nursing home placement" OR "old people's home*" OR "homes for the aged" OR "old age home" OR "old age homes" OR "housing for the elderly")
#11	**AB** ("elderly care institution" OR "nursing home*" OR "health care setting" OR "long term care facilities" OR "residential aged care" OR "care home*" OR "residential facilities" OR "homes for aged" OR "residential home*" OR "residential facilit*" OR "nursing home facility*" OR "nursing home care" OR "nursing home placement" OR "old people's home*" OR "homes for the aged" OR "old age home" OR "old age homes" OR "housing for the elderly")
#12	#9 OR #10 OR #11
#13	**(MH "Qualitative Studies+")**
#14	**TI** ("nursing methodology" OR "case study" OR "constant comparison" OR "content analysis" OR "descriptive study" OR "discourse analysis" OR ethnography OR exploratory OR feminist OR "focus group*" OR "grounded theory" OR hermeneutic OR interview OR narrative OR naturalistic OR "participant observation" OR phenomenology OR "qualitative method" OR "qualitative research" OR "qualitative study" OR "thematic analysis" OR "mixed method research" OR "mixed methods research")
#15	**AB** ("nursing methodology" OR "case study" OR "constant comparison" OR "content analysis" OR "descriptive study" OR "discourse analysis" OR ethnography OR exploratory OR feminist OR "focus group*" OR "grounded theory" OR hermeneutic OR interview OR narrative OR naturalistic OR "participant observation" OR phenomenology OR "qualitative method" OR "qualitative research" OR "qualitative study" OR "thematic analysis" OR "mixed method research" OR "mixed methods research")
#16	#13 OR #14 OR #15
#17	#4 AND #8 AND #12 AND #16
5. Search strategy for PsycINFO	
#1	**(DE "Dementia") OR (DE "Dementia with Lewy Bodies") OR (DE "Vascular Dementia") OR (DE "Alzheimer's Disease") OR (DE "Cognitive Impairment") OR (DE "Mild Cognitive Impairment")**
#2	**TI** (dement* OR "presenile dementia" OR "cognitive impairment" OR "cognitive dysfunction" OR "Alzheimer's disease" OR "Alzheimer's dementia" OR "AD" OR "mild cognitive impairment" OR "huntington disease" OR "lewy body" OR "chronic cerebrovascular" OR "parkinson disease dementia" OR "parkinson dementia" OR “lewy bodies” OR “lewy neurites” OR “dementia, vascular” OR “vascular dementia*”)
#3	**AB** (dement* OR "presenile dementia" OR "cognitive impairment" OR "cognitive dysfunction" OR "Alzheimer's disease" OR "Alzheimer's dementia" OR "AD" OR "mild cognitive impairment" OR "huntington disease" OR "lewy body" OR "chronic cerebrovascular" OR "parkinson disease dementia" OR "parkinson dementia" OR “lewy bodies” OR “lewy neurites” OR “dementia, vascular” OR “vascular dementia*”)

#4	#1 OR #2 OR #3
#5	**(DE "Nursing") OR (DE "Caregiving") OR (DE "Long Term Care") OR (DE "Management")**
#6	**TI** (nursing OR "practical nursing" OR "care, nursing" OR "management, nursing Care" OR "care, long-term" OR "long term care" OR care OR caring OR caregiving OR cargiving OR manag* OR "long-term care")
#7	**AB** (nursing OR "practical nursing" OR "care, nursing" OR "management, nursing Care" OR "care, long-term" OR "long term care" OR care OR caring OR caregiving OR manag* OR "long-term care")
#8	#5 OR #6 OR #7
#9	**(DE "Nursing Homes") OR (DE "Residential Care Institutions")**
#10	**TI** ("elderly care institution" OR "nursing home*" OR "health care setting" OR "long term care facilities" OR "residential aged care" OR "care home*" OR "residential facilities" OR "homes for aged" OR "residential home*" OR "residential facilit*" OR "nursing home facility*" OR "nursing home care" OR "nursing home placement" OR "old people's home*" OR "homes for the aged" OR "old age home" OR "old age homes" OR "housing for the elderly")
#11	**AB** ("elderly care institution" OR "nursing home*" OR "health care setting" OR "long term care facilities" OR "residential aged care" OR "care home*" OR "residential facilities" OR "homes for aged" OR "residential home*" OR "residential facilit*" OR "nursing home facility*" OR "nursing home care" OR "nursing home placement" OR "old people's home*" OR "homes for the aged" OR "old age home" OR "old age homes" OR "housing for the elderly")
#12	#9 OR #10 OR #11
#13	**DE "Focus Group" OR DE "Focus Group Interview" OR DE "Qualitative Methods" OR DE "Grounded Theory" OR DE "Interpretative Phenomenological Analysis" OR DE "Narrative Analysis" OR DE "Semi-Structured Interview" OR DE "Thematic Analysis"**
#14	**TI** ("nursing methodology" OR "case study" OR "constant comparison" OR "content analysis" OR "descriptive study" OR "discourse analysis" OR ethnography OR exploratory OR feminist OR "focus group*" OR "grounded theory" OR hermeneutic OR interview OR narrative OR naturalistic OR "participant observation" OR phenomenology OR "qualitative method" OR "qualitative research" OR "qualitative study" OR "thematic analysis" OR "mixed method research" OR "mixed methods research")
#15	**AB** ("nursing methodology" OR "case study" OR "constant comparison" OR "content analysis" OR "descriptive study" OR "discourse analysis" OR ethnography OR exploratory OR feminist OR "focus group*" OR "grounded theory" OR hermeneutic OR interview OR narrative OR naturalistic OR "participant observation" OR phenomenology OR "qualitative method" OR "qualitative research" OR "qualitative study" OR "thematic analysis" OR "mixed method research" OR "mixed methods research")
#16	#13 OR #14 OR #15
#17	#4 AND #8 AND #12 AND #16

1. Search strategy for Wan Fang	
#1	**主题:**(″痴呆″ OR ″老年痴呆″ OR ″血管性痴呆″ OR ″额颞叶性痴呆″ OR ″帕金森痴呆″ OR ″失智症″ OR ″认知症″ OR ″认知障碍″ OR ″阿尔茨海默″ OR ″阿尔兹海默″ OR ″Alzheimer″ OR ″AD″ OR ″轻度认知障碍″ OR ″MCI″ OR ″路易体痴呆″)
#2	**主题:**(″照护″ OR ″照顾″ OR ″护理″ OR ″管理″ OR ″照料″ OR ″照管″ OR ″照看″ OR ″看护″)
#3	**主题:**(″养老机构″ OR ″养老院″ OR ″护理之家″ OR ″幸福院″ OR ″老人院″ OR ″长期照护机构″ OR ″长期照顾机构″ OR ″长期护理机构″ OR ″长期看护机构″ OR ″敬老院″ OR ″安老院″ OR ″护理院″ OR ″老年人公寓″)
#4	**主题:**(″质性研究″ OR ″定性研究″ OR ″描述性研究″ OR ″民族志″ OR ″现象学″ OR ″扎根理论″ OR ″内容分析″ OR ″主题分析″ OR ″焦点小组″ OR ″解释学″ OR ″访谈″ OR ″叙述″ OR ″混合性研究″)
#5	#1 AND #2 AND #3 AND #4
2. Search strategy for CNKI	
#1	**SU** %= ’痴呆’ + ’老年痴呆’ + ’血管性痴呆’ + ’额颞叶性痴呆’ + ’帕金森痴呆’ + ’失智症’ + ’认知症’ + ’认知障碍’ + ’阿尔茨海默’ + ’阿尔兹海默’ + ’Alzheimer’ + ’AD’ + ’轻度认知障碍’ + ’MCI’ + ’路易体痴呆’
#2	**SU** %= ’照护’ + ’照顾’ + ’护理’ + ’管理’ + ’照料’ + ’照管’ + ’照看’ + ’看护’
#3	**SU** %= ’养老机构’ + ’养老院’ + ’护理之家’ + ’幸福院’ + ’老人院’ + ’长期照护机构’ + ’长期照顾机构’ + ’长期护理机构’ + ’长期看护机构’ + ’敬老院’ + ’安老院’ + ’护理院’ + ’老年人公寓’
#4	**SU** %= ’质性研究’ + ’定性研究’ + ’描述性研究’ + ’民族志’ + ’现象学’ + ’扎根理论’ + ’内容分析’ + ’主题分析’ + ’焦点小组’ + ’解释学’ + ’访谈’ + ’叙述’ + ’混合型研究’
#5	#1 AND #2 AND #3 AND #4
3. Search strategy for CBM	
#1	**″痴呆, 血管性″[不加权:扩展] OR ″阿尔茨海默病″[不加权:扩展] OR ″Lewy 体病″[不加权:扩展] OR ″阿尔茨海默病″[不加权:扩展] OR ″认知障碍″[不加权:扩展]**
#2	″痴呆″**[摘要:智能]** OR ″老年痴呆″[摘要:智能] OR ″血管性痴呆″[摘要:智能] OR ″额颞性痴呆″[摘要:智能] OR ″帕金森痴呆″[摘要:智能] OR ″失智症″[摘要:智能] OR ″认知症″[摘要:智能] OR ″认知障碍″[摘要:智能] OR ″阿尔茨海默″[摘要:智能] OR ″阿尔兹海默″[摘要:智能] OR ″Alzheimer″[摘要:智能] OR ″AD″[摘要:智能] OR ″轻度认知障碍″[摘要:智能] OR ″MCI″[摘要:智能] OR ″路易体痴呆″[摘要:智能]
#3	″痴呆″**[标题:智能]** OR ″老年痴呆″[标题:智能] OR ″血管性痴呆″[标题:智能] OR ″额颞性痴呆″[标题:智能] OR ″帕金森痴呆″[标题:智能] OR ″失智症″[标题:智能] OR ″认知症″[标题:智能] OR ″认知障碍″[标题:智能] OR ″阿尔茨海默″[标题:智能] OR ″阿尔兹海默″[标题:智能] OR ″Alzheimer″[标题:智能] OR ″AD″[标题:智能] OR ″轻度认知障碍″[标题:智能] OR ″MCI″[标题:智能] OR ″路易体痴呆″[标题:智能]
#4	#1 OR #2 OR #3

For peer review only

#5	**″护理″[不加权:扩展]**
#6	″照护″**[摘要:智能]** OR ″照顾″[摘要:智能] OR ″护理″[摘要:智能] OR ″管理″[摘要:智能] OR ″照料″[摘要:智能] OR ″照管″[摘要:智能] OR ″照看″[摘要:智能] OR ″看护″[摘要:智能]
#7	″照护″**[标题:智能]** OR ″照顾″[标题:智能] OR ″护理″[标题:智能] OR ″管理″[标题:智能] OR ″照料″[标题:智能] OR ″照管″[标题:智能] OR ″照看″[标题:智能] OR ″看护″[标题:智能]
#8	#5 OR #6 OR #7
#9	**″老人疗养院″[不加权:扩展]**
#10	″养老机构″**[摘要:智能]** OR ″养老院″[摘要:智能] OR ″护理之家″[摘要:智能] OR ″幸福院″[摘要:智能] OR ″老人院″[摘要:智能] OR ″长期照护机构″[摘要:智能] OR ″长期护理机构″[摘要:智能] OR ″长期照顾机构″[摘要:智能] OR ″长期看护机构″[摘要:智能] OR ″敬老院″[摘要:智能] OR ″安老院″[摘要:智能] OR ″护理院″[摘要:智能] OR ″老年人公寓″[摘要:智能]
#11	″养老机构″**[标题:智能]** OR ″养老院″[标题:智能] OR ″护理之家″[标题:智能] OR ″幸福院″[标题:智能] OR ″老人院″[标题:智能] OR ″长期照护机构″[标题:智能] OR ″长期护理机构″[标题:智能] OR ″长期照顾机构″[标题:智能] OR ″长期看护机构″[标题:智能] OR ″敬老院″[标题:智能] OR ″安老院″[标题:智能] OR ″护理院″[标题:智能] OR ″老年人公寓″[标题:智能]
#12	#9 OR #10 OR #11
#13	″质性研究″**[摘要:智能]** OR ″定性研究″[摘要:智能] OR ″描述性研究″[摘要:智能] OR ″民族志″[摘要:智能] OR ″现象学″[摘要:智能] OR ″扎根理论″[摘要:智能] OR ″内容分析″[摘要:智能] OR ″主题分析″[摘要:智能] OR ″焦点小组″[摘要:智能] OR ″解释学″[摘要:智能] OR ″访谈″[摘要:智能] OR ″叙述″[摘要:智能] OR ″混合型研究″[摘要:智能]
#14	″质性研究″**[标题:智能]** OR ″定性研究″[标题:智能] OR ″描述性研究″[标题:智能] OR ″民族志″[标题:智能] OR ″现象学″[标题:智能] OR ″扎根理论″[标题:智能] OR ″内容分析″[标题:智能] OR ″主题分析″[标题:智能] OR ″焦点小组″[标题:智能] OR ″解释学″[标题:智能] OR ″访谈″[标题:智能] OR ″叙述″[标题:智能] OR ″混合型研究″[标题:智能]
#15	#13 OR #14
#16	#4 AND #8 AND #12 AND #15
4. Search strategy for VIP	
#1	**M**=(″痴呆″ + ″老年痴呆″ + ″失智症″ + ″认知症″ + ″认知障碍″ + ″阿尔茨海默″ + ″阿尔兹海默″ + ″AD″ + ″轻度认知障碍″ + ″MCI″ + ″额颞叶性痴″ + ″血管性痴呆″ + ″帕金森痴呆″ + ″Alzheimer″ + ″路易体痴呆″) OR **R**=(″痴呆″ + ″老年痴呆″ + ″失智症″ + ″认知症″ + ″认知障碍″ + ″阿尔茨海默″ + ″阿尔兹海默″ + ″AD″ + ″轻度认知障碍″ + ″MCI″ + ″额颞叶性痴″ + ″血管性痴呆″ + ″帕金森痴呆″ + ″Alzheimer″ + ″路易体痴呆″)
#2	**M**=(″照护″ + ″护理″ + ″照顾″ + ″照料″ + ″照看″ + ″照管″ + ″看护″) OR **R**=(″照护″ + ″护理″ + ″照顾″ + ″照料″ + ″照看″ + ″照管″ + ″看护″)
#3	**M**=(″养老机构″ + ″养老院″ + ″护理之家″ + ″幸福院″ + ″老人院″ + ″敬老院″ + ″安老院″ + ″护理院″ + ″老年人公寓″) OR **R**=(″养老机构″ + ″养老院″ + ″护理之家″ + ″幸福院″ + ″老人院″ + ″敬老院″ + ″安老院″ + ″护理院″ + ″老年人

For peer review only

	公寓”)
#4	**M**=(”质性研究” + ”定性研究” + ”描述性研究” + ”民族志” + ”现象学” + ”扎根理论” + ”内容分析” + ”主题分析” + ”焦点小组” + ”解释学” + ”访谈” + ”叙述” + ”混合性研究”) OR **R**=(”质性研究” + ”定性研究” + ”描述性研究” + ”民族志” + ”现象学” + ”扎根理论” + ”内容分析” + ”主题分析” + ”焦点小组” + ”解释学” + ”访谈” + ”叙述” + ”混合性研究”)
#5	#1 AND #2 AND #3 AND #4

For peer review only

JBI Critical Appraisal Checklist for Qualitative Research

Reviewer___________________________________Date__________________________

Author ___________________________________Year________Record Number______

	Yes	No	Unclear	Not applicable
1. Is there congruity between the stated philosophical perspective and the research methodology?	☐	☐	☐	☐
2. Is there congruity between the research methodology and the research question or objectives?	☐	☐	☐	☐
3. Is there congruity between the research methodology and the methods used to collect data?	☐	☐	☐	☐
4. Is there congruity between the research methodology and the representation and analysis of data?	☐	☐	☐	☐
5. Is there congruity between the research methodology and the interpretation of results?	☐	☐	☐	☐
6. Is there a statement locating the researcher culturally or theoretically?	☐	☐	☐	☐
7. Is the influence of the researcher on the research, and vice- versa, addressed?	☐	☐	☐	☐
8. Are participants, and their voices, adequately represented?	☐	☐	☐	☐
9. Is the research ethical according to current criteria or, for recent studies, and is there evidence of ethical approval by an appropriate body?	☐	☐	☐	☐
10. Do the conclusions drawn in the research report flow from the analysis, or interpretation, of the data?	☐	☐	☐	☐

Overall appraisal: Include ☐ Exclude ☐ Seek further info ☐

Comments (Including reason for exclusion)

____

____

____

JBI QARI Data Extraction Tool for Qualitative Research

Reviewer ______________________ Date ______________________

Author ______________________ Year ______________________

Journal ______________________ Record Number ______________________

Study Description

Methodology

Method

Phenomena of interest

Setting

Geographical

Cultural

Participants

Data analysis

Authors conclusions

Comments

Complete Yes ☐ No ☐

Copyright © The Jo0anna Briggs Institute 2014

Findings	Illustration form Publication (page number)	Evidence		
		Unequivocal	Credible	Unsupported

Extraction of findings complete Yes ☐ No ☐

Copyright © The Jo0anna Briggs Institute 2014

Reporting checklist for protocol of a systematic review and meta analysis.

Based on the PRISMA-P guidelines.

Instructions to authors

Complete this checklist by entering the page numbers from your manuscript where readers will find each of the items listed below.

Your article may not currently address all the items on the checklist. Please modify your text to include the missing information. If you are certain that an item does not apply, please write "n/a" and provide a short explanation.

Upload your completed checklist as an extra file when you submit to a journal.

In your methods section, say that you used the PRISMA-Preporting guidelines, and cite them as:

Moher D, Shamseer L, Clarke M, Ghersi D, Liberati A, Petticrew M, Shekelle P, Stewart LA. Preferred Reporting Items for Systematic Review and Meta-Analysis Protocols (PRISMA-P) 2015 statement. Syst Rev. 2015;4(1):1.

For peer review only

		Reporting Item	Page Number
**Title**			
Identification	#1a	Identify the report as a protocol of a systematic review	Lines 1-2
Update	#1b	If the protocol is for an update of a previous systematic review, identify as such	n/a

**Registration**			
	#2	If registered, provide the name of the registry (such as PROSPERO) and registration number	Lines 58-60
**Authors**			
Contact	#3a	Provide name, institutional affiliation, e-mail address of all protocol authors; provide physical mailing address of corresponding author	Lines 4-19
Contribution	#3b	Describe contributions of protocol authors and identify the guarantor of the review	Lines 282-288
**Amendments**			
	#4	If the protocol represents an amendment of a previously completed or published protocol, identify as such and list changes; otherwise, state plan for documenting important protocol amendments	n/a
**Support**			
Sources	#5a	Indicate sources of financial or other support for the review	Lines 280-281
Sponsor	#5b	Provide name for the review funder and / or sponsor	n/a
Role of sponsor or funder	#5c	Describe roles of funder(s), sponsor(s), and / or institution(s), if any, in developing the protocol	n/a
**Introduction**			

For peer review only

Rationale	#6	Describe the rationale for the review in the context of what is already known	Lines 74-139
Objectives	#7	Provide an explicit statement of the question(s) the review will address with reference to participants, interventions, comparators, and outcomes (PICO)	Lines 140-146
**Methods**			
Eligibility criteria	#8	Specify the study characteristics (such as PICO, study design, setting, time frame) and report characteristics (such as years considered, language, publication status) to be used as criteria for eligibility for the review	Lines 156-173
Information sources	#9	Describe all intended information sources (such as electronic databases, contact with study authors, trial registers or other grey literature sources) with planned dates of coverage	Lines 174-180
Search strategy	#10	Present draft of search strategy to be used for at least one electronic database, including planned limits, such that it could be repeated	Supplementary File 1
Study records - data management	#11a	Describe the mechanism(s) that will be used to manage records and data throughout the review	Lines 197-201
Study records - selection process	#11b	State the process that will be used for selecting studies (such as two independent reviewers) through each phase of the review (that is, screening, eligibility and	Lines 196-224

For peer review only

		inclusion in meta-analysis)	
Study records - data collection process	#11c	Describe planned method of extracting data from reports (such as piloting forms, done independently, in duplicate), any processes for obtaining and confirming data from investigators	Lines 225-241
Data items	#12	List and define all variables for which data will be sought (such as PICO items, funding sources), any pre-planned data assumptions and simplifications	Lines 228-233
Outcomes and prioritization	#13	List and define all outcomes for which data will be sought, including prioritization of main and additional outcomes, with rationale	Lines 161-164
Risk of bias in individual studies	#14	Describe anticipated methods for assessing risk of bias of individual studies, including whether this will be done at the outcome or study level, or both; state how this information will be used in data synthesis	Lines 212-224
Data synthesis	#15a	Describe criteria under which study data will be quantitatively synthesised	n/a
Data synthesis	#15b	If data are appropriate for quantitative synthesis, describe planned summary measures, methods of handling data and methods of combining data from studies, including any planned exploration of consistency (such as I2, Kendall's τ)	n/a
Data synthesis	#15c	Describe any proposed additional analyses (such as	n/a

For peer review only

		sensitivity or subgroup analyses, meta-regression)	
Data synthesis	#15d	If quantitative synthesis is not appropriate, describe the type of summary planned	Lines 242-260
Meta-bias(es)	#16	Specify any planned assessment of meta-bias(es) (such as publication bias across studies, selective reporting within studies)	Lines 71-72
Confidence in cumulative evidence	#17	Describe how the strength of the body of evidence will be assessed (such as GRADE)	Lines 261-270

None The PRISMA-P elaboration and explanation paper is distributed under the terms of the Creative Commons Attribution License CC-BY. This checklist can be completed online using https://www.goodreports.org/, a tool made by the EQUATOR Network in collaboration with Penelope.ai

For peer review only
